# A Systematic Review of Biochar Research, with a Focus on Its Stability *in situ* and Its Promise as a Climate Mitigation Strategy

**DOI:** 10.1371/journal.pone.0075932

**Published:** 2013-09-30

**Authors:** Noel P. Gurwick, Lisa A. Moore, Charlene Kelly, Patricia Elias

**Affiliations:** 1 Smithsonian Environmental Research Center. Edgewater, Maryland, United States of America; 2 Environmental Defense Fund, San Francisco, California, United States of America; 3 Western Carolina University, North Carolina, United States of America; 4 Union of Concerned Scientists, Washington, District of Columbia, United States of America; The Ohio State University, United States of America

## Abstract

**Background:**

Claims about the environmental benefits of charring biomass and applying the resulting “biochar” to soil are impressive. If true, they could influence land management worldwide. Alleged benefits include increased crop yields, soil fertility, and water-holding capacity; the most widely discussed idea is that applying biochar to soil will mitigate climate change. This claim rests on the assumption that biochar persists for hundreds or thousands of years, thus storing carbon that would otherwise decompose. We conducted a systematic review to quantify research effort directed toward ten aspects of biochar and closely evaluated the literature concerning biochar's stability.

**Findings:**

We identified 311 peer-reviewed research articles published through 2011. We found very few field studies that addressed biochar's influence on several ecosystem processes: one on soil nutrient loss, one on soil contaminants, six concerning non-CO_2_ greenhouse gas (GHG) fluxes (some of which fail to support claims that biochar decreases non-CO_2_ GHG fluxes), and 16–19 on plants and soil properties. Of 74 studies related to biochar stability, transport or fate in soil, only seven estimated biochar decomposition rates *in situ*, with mean residence times ranging from 8 to almost 4,000 years.

**Conclusions:**

Our review shows there are not enough data to draw conclusions about how biochar production and application affect whole-system GHG budgets. Wide-ranging estimates of a key variable, biochar stability *in situ*, likely result from diverse environmental conditions, feedstocks, and study designs. There are even fewer data about the extent to which biochar stimulates decomposition of soil organic matter or affects non-CO_2_ GHG emissions. Identifying conditions where biochar amendments yield favorable GHG budgets requires a systematic field research program. Finally, evaluating biochar's suitability as a climate mitigation strategy requires comparing its effects with alternative uses of biomass and considering GHG budgets over both long and short time scales.

## Introduction

In the face of the dual challenge to mitigate global climate change and ensure food security for a growing global population, crop management strategies that build soil organic matter (SOM) have received considerable attention [1]–[4]. Because photosynthesis converts CO_2_ to organic carbon, increases in plant carbon stocks reduce atmospheric CO_2_ concentrations. When plant biomass does not rapidly decompose but instead is stored in wood or increased SOM, it keeps carbon out of the atmosphere. Increased SOM also enhances soil fertility and water holding capacity [4], [5]. As a strategy for climate change mitigation, a key weakness of enhanced soil storage of carbon is the susceptibility of SOM to decomposition and re-release of CO_2_ to the atmosphere [6], [7].

A related carbon storage proposal that gained considerable attention at the beginning of the 21^st^ century calls for heating biomass in the absence of oxygen (pyrolysis) and applying the resulting carbonized material to agricultural or forest soils [8]–[10]. The solid product of pyrolysis, called “biochar” in the context of climate change mitigation, is highly heterogeneous material with chemical composition that varies widely depending on feedstock and pyrolysis conditions [11]. The scale of biochar production and application being discussed is enormous. For example, Matovic [12] calculated that charring and burying 10% of global net primary productivity each year would offset the current annual increase in atmospheric CO_2_.

This discussion has reached far beyond the academic literature, to highly visible media outlets [13], books about solutions to address climate change [14], and specific policy proposals for carbon offsets [15]. The California Energy Commission's Public Interest Energy Research Program has supported work to describe biochar projects that could qualify as greenhouse gas offsets, which would effectively trade carbon sequestration by biochar for emissions of fossil fuel derived CO_2_ that would otherwise be limited by regulation [16]. With this funding, the Climate Trust, a US-based non-profit that specializes in “climate solutions for governments, utilities, and large businesses” has written that “at its maximum sustainable potential, biochar could” reduce annual global GHG emissions by 12% [16]; to arrive at this estimate, they assumed that 80% of biochar carbon persists in soil after 100 years. The International Biochar Initiative (IBI) promotes ubiquitous use of biochar as a soil amendment, advocates for inclusion of provisions favorable to biochar use in national and global climate mitigation policies, promotes biochar commercialization, and aspires to a global system that sequesters 2.2 Gt C/yr by 2050 [17]. Addressing international climate change policy, IBI has urged that the technical advisory body of the UN Framework Convention on Climate Change incorporate biochar within the work program on mitigation in the agricultural sector [18]. In April 2013, IBI also reported that a methodology for using biochar amendments as an offset protocol was soon to be submitted for consideration by the American Carbon Registry, which plays a key role in establishing protocols for carbon markets [19]. Finally, proponents of biochar use often suggest it has numerous benefits, as illustrated in IBI's description of biochar's benefits: “Biochar is uniquely positioned to aid in these critical overlapping arenas [of climate mitigation and adaptation and food security] by building soil carbon sinks and mitigating climate change while also enhancing soil quality and resilience to drought and certain diseases [18].”

Underlying the idea that incorporating biochar in soil effectively mitigates climate change is the argument that it resists degradation and will persist in soil for hundreds or thousands of years [14], [20], [21]. However, claims about biochar's stability in soil rest most frequently on observations of old charcoal in Amazonian and other soils [22]. The presence of ancient charcoal in soil shows only that some old charcoal persists; it could be a small fraction of a much larger stock of since-decayed charred biomass [11]. Even if it is the majority of the original stock, persistence of biochar carbon could well be context-dependent, remaining stable in some soils and climate regimes and not in others. The biochar systems that are being proposed for climate mitigation would apply highly variable biochars – produced from diverse feedstocks and under varying pyrolysis conditions – to diverse soils under a wide range of environmental conditions. What do empirical data indicate about biochar's stability *in situ*?

Although understanding biochar stability is critical to quantifying the impact of biochar amendments on net greenhouse gas (GHG) emissions to the atmosphere, it is not sufficient. Additional factors that need to be considered include emissions associated with growing, harvesting and transporting feedstock; and with biochar production and application to soil. Life cycle analysis (LCA) integrates these varied sources of emissions associated with biochar amendments to soil to understand the system-level impacts on terrestrial carbon stocks and atmospheric GHG concentrations [23]. The confidence we should place in LCA results is directly related to availability of data used to estimate model inputs.

Given the emphasis that biochar proponents have placed on biochar stability in soil, we focused this review on that aspect of biochar, but reported benefits of biochar also include increased soil fertility and water holding capacity, increased crop production, and remediation of contaminated soils. The literature provides some support for these claims [24]–[30], but there is no evidence that all these claims have been tested across a broad enough variety of landscapes, climates, and management systems to draw robust conclusions [31]. Furthermore some researchers have cautioned that biochar could have adverse effects by releasing toxic substances such as heavy metals into soil or reducing the efficacy of pesticides [32]. How frequently these unintended consequences of biochar amendment occur is unclear. To determine whether there is sufficient research to support adoption of biochar systems, there is a strong need to review the peer-reviewed literature with respect to the multiple benefits and risks biochar may deliver.

In this paper, we systematically review and organize the biochar literature towards the following three objectives: (1) quantitatively characterize the research effort that has been directed towards a variety of biochar attributes (*e.g*., production processes, effects on soil or plant production, etc.); (2) provide a more focused characterization of the biochar literature concerning biochar stability; and (3) identify and review field studies that have calculated biochar stability. We also consider the broader implications of existing data for implementing biochar systems.

## Methods

### Searching

We searched for articles using the terms “biochar” and “bio-char” on the Web of Science (in the ‘topic’ field) and Agricola databases for papers published prior to January 1, 2012. Because the biochar literature is so diverse and the term “biochar” is relatively new, we supplemented our search with citations in recent studies. To further ensure that we had assembled a comprehensive list of studies, we asked researchers in a leading biochar research group to review and suggest additions to our database.

### Screening

We excluded search results that were published in languages other than English or for which only an abstract was available, and then characterized each remaining search result as one of five publication types. “Primary research” papers appeared in the peer-reviewed literature and reported original data or results from observations, experiments, or models. “Methods” papers evaluated or described an investigative technique for studying biochar. “Review” papers summarized understanding of biochar but did not report new data. Our search also captured “other” publication types such as news stories, book chapters, extension newsletters, editorial notes, and letters-to-the-editor. Finally, we characterized as “incidental” any publication that did not concern biochar in any meaningful way; many publications, for example, listed but did not analyze or describe the products of pyrolysis, one of which is biochar. Throughout our screening process, we noted the number of publications identified during our search, the number of publications excluded in each stage, and the reasons for those exclusions, following the guidelines set forth in the Preferred Reporting Items for Systematic Reviews and Meta-Analyses (PRISMA) Statement (Figure S1) [33].

### Objectives 1 and 2 – characterizing the literature

To achieve our first objective – a quantitative characterization of the biochar literature as a whole – we applied a second sorting process to the publications identified as primary research. Based on an initial reading of these papers, we identified ten topic areas and assigned each paper to all topic areas that applied. We also noted whether each study had a laboratory and/or *in situ* component. The ten topic areas were:

1. *Stability, transport, or fate of biochar or soil carbon* – Studies that described changes in the physical and/or chemical properties of biochar (or soil organic matter mixed with biochar) over time; measured or inferred changes in a system's carbon balance after the addition of biochar; or described the amount or location of biochar in a soil or landscape at a single time point.
2. *Model and/or life cycle analyses of biochar systems –* Studies that calculated the economic, energy and/or climate change mitigation potential of biochar production systems.
3. *Influence of biochar on non-CO_2_ trace gas emissions from soil –* Studies that reported rates of methane and/or nitrous oxide emissions from soils to which biochar had been added.
4. *Soil nutrients –* Studies that reported nutrient levels in soils amended with biochar.
5. *Plant responses –* Studies that reported responses, such as yield or nutrient status, of plants grown on soils amended with biochar.
6. *Soil biology* – Studies that described or reported changes in soil microbes, fungi, earthworms, or other soil fauna on biochar or in soil amended with biochar.
7. *Soil properties* – Studies that reported, for example, the pH, bulk density, water holding capacity, or cation exchange capacity of soils amended with biochar.
8. *Nutrient loss (N, P, K)* – Studies that reported loss of nutrients, for example via leaching or gaseous emissions, from soils amended with biochar.
9. *Biochar production and analysis* – Studies that described biochar production processes and/or characterized the physical or chemical properties of biochar.
10. *Influence on contaminants –* Studies that described the effects of biochar on the levels or mobility of contaminants such as lead, arsenic, pesticides, and herbicides in water or soil.

To achieve our second objective – a quantitative characterization of the literature related to the stability of biochar – we focused on primary research articles that addressed the stability, transport, or fate of biochar or soil carbon (topic area 1, above). We sorted these studies in three stages. First, because estimating biochar stability by definition requires measuring a change in biochar amount or characteristics over time, we divided studies that took measurements at one time point from studies that tracked changes over time. We then divided the studies that tracked changes over time into field versus lab studies. Although laboratory incubations are valuable because they can test *relative* stability of different substrates and the mechanisms that control decomposition [11], [34], [35], we focused on field studies because they provide unique and essential data for deriving realistic estimates of biochar stability under real-world conditions. Finally, we divided the field studies into experiments that did or did not calculate a measure of biochar stability, such as mean residence time or turnover time.

### Objective 3 – Reviewing scientific understanding of biochar stability

We looked closely at each study in this last group to address our third objective, which was to ascertain what can be said with confidence about the stability of biochar under field conditions. To allow a synthetic view of findings from these key studies, we noted for each study its location, ecosystem, type of biochar used, method of biochar application to the study site, and numerical estimate of biochar stability. Because converting among measures of stability (*e.g.*, half-life, turnover time, mean residence time) requires assumptions about the shape of the decay curve, we did not convert all these measurements into a “common currency” and instead report numbers as they appeared in each paper.

## Results

Our initial search yielded 472 unique publications (Figure S1). We excluded 161 of these from further analysis either because full English text was unavailable (three publications) or because the publication was not primary research (158 publications, including 63 non-peer-reviewed (“other”) publications, 53 literature reviews, 27 papers with only incidental mention of biochar, 14 articles describing new methods, and one paper captured by our search because it used the unrelated term “biocharacters”).

**Figure 1:**
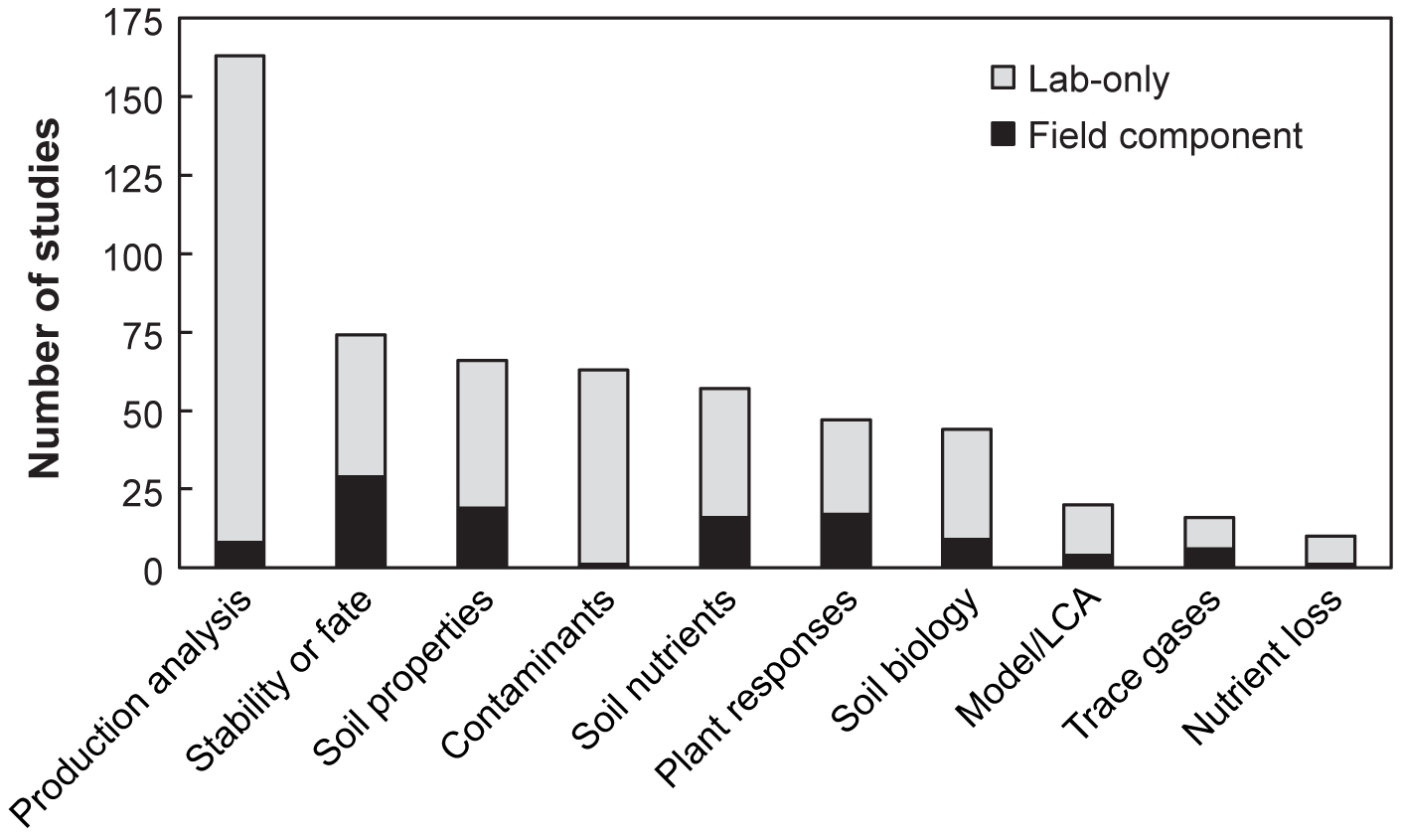
Number of primary research articles addressing each topic area. The dark shaded area indicates the studies that included a field component.

Each of the remaining 311 peer-reviewed articles (Figure S2) described primary research in at least one of the ten topic areas (Figures 1 and S3). Most commonly, these studies tested pyrolysis techniques used to produce char and characterized the resulting materials. Full life cycle analysis of biochar systems and the effects of biochar on non-CO_2_ trace gas and soil nutrient fluxes have received the least attention (Figure 1).

*In situ* research investigating the influence of biochar on nutrient loss and contaminants was almost non-existent (1 study each) (black bars in Figure 1). Field research was more common for studies of plant responses, soil nutrients, and soil properties (16–19 studies each, Figure 1). Of the 20 model/LCA studies, only four used field measurements, and of 74 original research papers that addressed biochar stability or fate, 29 included a field component.

Seventy-four studies addressed some aspect of the stability, transport, or fate of biochar in soil (Figures S2 and S3), with 60 measuring system change over time. Of those, 18 articles reported results from a field experiment. Seven of those field studies provided some quantitative estimate of biochar stability derived from measurements of biochar stocks or soil respiration (Table 1). Three of the seven studies experimentally added biochar to soil, and four used naturally-occurring biochar from fire at sites differing in fire frequency over a number of years.

**Table 1:** Field experiments estimating biochar stability.

Study	Location (ecosystem)	Biochar source and application method	Study design	Biochar loss rate (years)
Major et al. [48]	Colombia (savanna)	Charred mango wood disked into soil	Measured soil respiration and leaching for 2 years after biochar addition	MRT 3,624
Haefele et al. [58]	Thailand and the Philippines (rice paddies)	Charred rice husks tilled into soil	Measured biochar C for 3 years after biochar addition	MRT >1,000
Knoblauch et al. [51]	Los Baños, Philippines (rice paddies)	Charred rice husks tilled into soil	Measured soil CO_2_ and CH_4_ emissions for 3 months immediately and 2 years after biochar addition	MRT “several hundred if not thousands”
Cheng et al. [68]	Eastern North America (various)	Collected from soils at historic charcoal furnaces	Compared C content of old charcoal to that of charcoal produced in reconstructed furnaces	22% of biochar C lost in 130
Hammes et al. [47]	Russia (steppe)	Naturally-occurring fire	Measured black carbon stocks at a 100-year fire suppression site	Turnover time 293
Bird et al. [50]	Zimbabwe (savanna)	Naturally-occurring fire	Measured charcoal and oxidation-resistant elemental carbon (OREC) abundance at a 50-year fire suppression site	Half-life “considerably <50 years” (charcoal) and <100 years (OREC)
Nguyen et al. [49]	Kenya (cropland)	Slash-and-burn conversion from forest to cropland	Measured black carbon stocks along a 100-year chronosequence	MRT 8.3

The locations, methods, and results of the seven experiments that measured or estimated biochar stability in a field setting. MRT is mean residence time. Assuming a steady decomposition rate, the results of Cheng et al. [68] imply a turnover time of 565 years. However, decomposition tends to slow over time, so turnover time is likely longer.

The seven studies were diverse in numerous respects (Table 1). They included temperate and tropical ecosystems (*e.g.*, Eastern North America, Philippines, Colombia) and applied biochar produced from a variety of feedstocks (*e.g.*, mango wood, rice husks, char from forest fires). These studies also used different analytical approaches. For example, some measured soil CO_2_ evolution for several years following char addition; others measured the amount of char in soil along a fire chronosequence at different times following the initiation of fire suppression, or after deliberate biochar addition at a site.

Four studies reported mean residence time (MRT) of biochar (ranging from ∼8 to ∼3,600 yrs), one reported turnover time (∼300 yrs), one reported half-life (“considerably <50 years”), and one reported the percentage of original biochar lost over 130 years (22%) (Table 1).

Despite our efforts to ensure that we had a comprehensive set of studies, it is of course possible that we missed some relevant papers published before “biochar” became widely used. However, comparison with a recent meta-analysis of biochar's effects on nutrient cycling and plant productivity suggests we captured the vast majority of important publications. Biederman and Harpole [36] included “charcoal” and “black carbon” in their search terms, which we did not, and found 114 papers through 2012 and 84 papers through 2011 (our cutoff). By comparison, of the 311 peer-reviewed publications we found through 2011, 115 fell within topic areas 4–7 (which encompass the variables examined by Biederman and Harpole), and these 115 included the large majority of the 84 papers included in their meta-analysis. Despite Biederman and Harpole's more expansive search terms, they found fewer, not more, references than we did in a comparable time frame, and even adding an additional year (2012) did not exceed the number of references we identified in their topic areas. The similar numbers of papers captured by the two studies may reflect our use of citations from recent studies and papers from the database of a leading biochar research laboratory, although these sources accounted for a relatively small proportion of the papers we reviewed. Although our criteria for excluding and categorizing studies differed from theirs, because the studies had different objectives, this comparison strongly suggests that adding additional search terms would not significantly have expanded the set of literature we analyzed, nor the primary patterns we found. Consistent with this conclusion, we conducted our study iteratively, and the patterns we discerned based on early analyses did not change as we added more papers and refined our exclusion and categorization criteria.

## Discussion

### Making decisions about biochar systems

At first glance, our review might suggest a robust literature on biochar (311 primary research articles; Figure 1), but information critical to evaluating biochar's influence on ecosystem properties and its fate in the environment is actually quite scarce and uneven across topic areas. The literature is dominated by studies of biochar production and material properties. This understanding is essential for developing production facilities, and may in the future become relevant for understanding biochar's influence on ecosystem services, as relationships among biochar chemistry and physical processes and chemical reactions in the soil system become better understood. In two categories of clear interest from an ecosystem perspective – nutrient loss and contaminants – we found only one field study each. Other categories have more studies, but even in those cases critical questions have not been addressed. For example, Vaccari et al. [37] documented as much as a 2°C increase in soil temperatures for soils amended with biochar using rotary tillage, suggesting that biochar amendment to soil is likely to change albedo significantly. Decreased albedo would increase soil temperature, which has been shown to accelerate soil respiration and affect soil nutrient availability, ecosystem water dynamics, species composition, and growing season length [38]–[40]. But of 19 field studies that addressed soil physical properties, only this one addressed albedo. Interrogating the literature within the broad topic areas we used is likely to yield many instances such as this, where studies needed to answer more specific questions are scarce or absent. The database we produced (Figure S3), with studies grouped into descriptive categories, provides rapid access to the relevant literature for scientists and land managers with specific questions in mind.

Our systematic review of original research on biochar revealed two features of this relatively young field that complicate efforts to make science-based decisions about biochar systems. First, the biochar literature lacks standardized nomenclature and analytical methods, reflecting the varied traditions of bioenergy engineering, agronomy, climate science, soil science and geology – all disciplines that have begun using the term “biochar” instead of charcoal or black carbon. Bringing multiple perspectives to bear on challenging problems often yields insights that might be missed in the absence of an interdisciplinary approach [41], [42], but the absence of standardized vocabulary and methods has complicated efforts to compare results among studies and distill the implications for policy and management. Second, laboratory studies dominate the biochar literature. Data from laboratory studies could be valuable for investigating mechanisms that control biochar behavior in the environment and its effects on ecosystem services [11], [34], [35]. However, whole ecosystem experiments have proven critical to testing policy-relevant hypotheses in numerous cases including the response of lakes to nutrient additions [43], the influence of acid precipitation on forests [44], and the response of terrestrial ecosystems to rising CO_2_ levels [45], [46]. In the absence of ecosystem-level experiments, the consequences of management actions are largely speculative, making it hard to justify specific policies. Plant-soil ecosystems are complex, with interactions and feedbacks that lab experiments do not fully mimic, and there is no reason to suppose that field experiments are any less essential in evaluating whole system responses to biochar additions than they have been for evaluating impacts of other anthropogenic activities.

### How stable is biochar in the field?

Proponents of biochar use have claimed it is long-lasting [14], [21], but very few data are available to evaluate the stability of biochar *in situ*. Only seven of the primary research papers we identified reported field investigations of biochar stability in soil, and their estimates of stability – although not easily and directly comparable – spanned three orders of magnitude, from years to millennia (Table 1). Moreover, only one of those studies quantified the uncertainty of the results: Hammes et al. [47] calculated that the turnover time of biochar in their study ranged from 182 to 541 years. Two studies calculated mean residence time via first-order decay models but did not discuss the uncertainty in the calculations [48], [49]. Other researchers noted “comparatively large” uncertainties [50], [51] and numerous investigators have cautioned that stability must be better understood [52]–[55].

Given the ubiquitous discussion of biochar as an extremely passive carbon pool, we asked whether we should discount the two studies reporting most rapid loss: <10 years mean residence time [49] and “considerably <50 years” half-life [50]. In both studies, the loss estimates include physical transport, so rates of microbial decomposition of biochar to CO_2_ may be lower than the reported loss rates, but in neither case are there data to estimate how much of the loss resulted from transport vs. decomposition. Nguyen et al. [49] used two different methods to estimate biochar abundance in the top 10 cm of soil at 9 sites along a fire chronosequence in Kenya and observed a 70% decrease in biochar content during the first 30 years. The site was relatively flat, and the authors therefore suggested that surface flow did not dominate biochar loss from the site. However, they noted that illuviation to deeper soil horizons may have been significant. Yanai et al. [56] urged caution when inferring organic matter responses to disturbance from soils collected along chronosequences because the nature of disturbance (such as logging techniques – horse vs. tractor) can change through time, so time *of* disturbance is difficult to separate from time *since* disturbance (the independent variable of interest). Nguyen et al. collected soils from fields where forest was cleared using slash-and-burn followed by plowing to 0.1–0.12 m depth, at eight points in time over 100 years. It is possible that different amounts of biomass had accumulated at different points in time, or that clearing methods were not identical, but burning, plowing, and plow depth were consistent across the chronosequence. Bird et al. [50] measured charcoal content in the upper 5 cm of soil from plots in Zimbabwe; some plots have a fire return interval of 1 to 5 years, while in others fire had been suppressed since 1947. Like Nguyen et al. [49], Bird et al. [50] noted that some degradation processes may have altered large particles to small particles which then moved beneath the shallow sampling depth. On balance these studies do not appear any less credible than the other three [47], [57], [58] that estimated biochar stability by measuring changes in biochar content in soil (Table 1). These findings caution against assuming that biochar persists 100 years or more, a value used in some LCA models [59], [60].

What might account for the wide variation in field-based estimates of biochar stability? These field experiments were conducted in a variety of ecosystems on several continents, leading to large variation in conditions such as temperature, moisture and microbial communities, all of which act on the biochar in each study (Table 1). The experiments also used different biochar feedstocks and pyrolysis conditions such as temperature, duration, and oxygen content, all of which affect biochar properties and hence stability [11]. Production methods included vegetation fires, historical kilns, carefully regulated commercial or laboratory reactor vessels, and simply piling biomass on top of a burning chamber and waiting for the pile to turn black.

This variation in experimental materials and conditions is a valuable feature of field-based studies of biochar. After all, biochar systems would be implemented in different ecosystems using a greater variety of biochars and methods than were reported in the seven field studies we identified. Similarly, the potential diversity of feedstocks and conditions that could be used is greater than represented in these field studies, as evidenced by the broader range of experimental conditions represented in the 311 primary research articles included in our review. For example, biochar feedstock could include animal waste [61], [62], agricultural waste [63], [64], and natural vegetation [65], [66]. Studies to date begin to establish the range of variation in biochar stability but do not go very far towards explaining it. As this young field begins to mature, field-based studies conducted across sites that vary systematically with respect to key variables such as temperature and moisture, and that span the full range of variation, combined with laboratory experiments, should help establish empirical understanding of why biochar stability ranges so widely and project how biochar might behave in a given setting [67].

### Limitations on estimating whole-system GHG balance

Ultimately, the suitability of biochar as a climate mitigation tool will depend on more than just biochar stability. In the field, biochar additions may influence plant growth and associated carbon uptake; decomposition of native soil organic matter; and emissions of non-CO_2_ greenhouse gases from soil, resulting from either changes in soil moisture or nutrient availability. Prior to applying biochar to soil, biomass harvest (or collection) and transport, and biochar production and field application influence GHG emissions. While it is neither necessary nor realistic to measure or estimate all aspects of a GHG budget with equal precision, and some terms may reasonably be judged *de minimus*, all elements do need to be at least considered in assessing the GHG and climate change mitigation potential of interventions like biochar addition to soil. In addition, using very different approaches to measure key terms of the GHG budget hampers confidence in comparisons of whole system GHG balances. For example, biochar stability estimated as mean residence time from changes in biochar stocks [47], [49], [50], [58], [68] is difficult to compare directly with stability estimated by soil CO_2_ emissions [48], [51]. Finally, choosing the best climate change mitigation strategy requires comparing total system emissions from a biochar-based system against emissions associated with alternative management strategies such as combusting biomass for energy or leaving biomass in the field.

Very few studies have reported the information needed to estimate how whole ecosystem GHG budgets respond to biochar additions, and rarely-studied components of the GHG budget (like non-CO_2_ emissions) sometimes respond to biochar amendment in ways that aggravate rather than mitigate climate change. We found only one field study that attempted to construct a whole system carbon budget of a plant-soil system in response to biochar additions vs. leaving the biomass in place [51]. We found six field studies of the effects of biochar on non-CO_2_ GHG emissions. Three found no significant effect of biochar on N_2_O emissions [29], [69], [70], contrary to common assertions that biochar decreases N_2_O flux [21], [60]. Two found no effect on CH_4_ emissions [69], [70], and one measured an increase in CH_4_ emissions [71]. Carbon additions to soil are well-known to stimulate decomposition of native soil carbon [72]–[75], and Wardle et al. [76] reported a “priming” effect of biochar, in which biochar amendment catalyzed decomposition of existing soil organic carbon (but see [77] and [78]). Whole system analysis of bioenergy crop production has included vigorous debate about GHG emissions associated with indirect land use change (ILUC) [79]–[81], and ILUC could affect evaluation of biochar systems compared to alternative biomass fates. For example, Roberts et al. [23] showed that ILUC can reduce or even reverse the climate mitigation potential of biochar systems.

Comprehensive comparisons of GHG emissions among management approaches are typically achieved through an LCA, and we found nine such studies in our review. These studies might appear to provide the kind of comparisons we need – taking into account the many potential sources of GHG emissions associated with implementing a biochar system – but in fact they face the data availability constraints discussed in this review. In addition, LCAs to date have considered only a very limited number of alternative biomass management options. Of the LCA studies included in our systematic review, only four considered management options besides pyrolysis as a basis for comparison [23], [59], [60], [82]. Two of these analyses compared biomass combustion for energy production to biomass conversion to biochar, and found that in some circumstances biomass combustion avoided more GHG emissions than the biochar system [23], [60]. Most of the LCAs we captured in our review cautioned that their default assumptions about biochar stability needed further testing and/or that their results were sensitive to their choice of biochar stability [52]–[55], [59], [60], [82], [83].

Evaluating the consequences of alternative fates for biomass takes on added weight when we consider that different management options lead to varied temporal patterns of carbon storage and release. Even in cases in which biochar additions to soil lead to GHG benefits over hundreds of years compared to alternative biomass management scenarios, approximately 50% of the biomass carbon will be released to the atmosphere as CO_2_ over the short term, either vented during pyrolysis or emitted when the other pyrolysis products such as bio-oil and syngas are combusted for energy [60]. In contrast, the half-life of uncharred wood of northern temperate tree species ranges from 6.8 to 150 years [84], [85], and wood decomposition proceeds gradually over that time period [85], [86], avoiding a large, near-term pulse of CO_2_ to the atmosphere. Similarly, most wood products are long-lived. For example, wood used for single-family housing in the US has a half-life of approximately 80 years [87], again avoiding large pulses of CO_2_ to the atmosphere on time scales of weeks to years, although CO_2_ release associated with harvest and wood processing need to be taken into account. Therefore, in many cases charring woody biomass would lead to greater radiative forcing in the short term than would leaving it to decompose or using it in long-lived wood products. Reducing GHG emissions in the short-term will lessen the unprecedented rate of climate change, facilitating to some extent the ability of ecosystems and human institutions to adapt.

We are not the first to caution that many claims about biochar are overly enthusiastic. For example, Kookana et al. [32] called for a more balanced evaluation of biochar's potential adverse environmental impacts, particularly a reduction in agrochemical effectiveness and the introduction of soil contaminants. Likewise, Jeffery et al. [31] called on the research community to “think ‘outside of the pot’” and criticized a recent meta-analysis [36] for failing to acknowledge important limitations (including a reliance on very short-term studies to infer long-term carbon storage) and over-stating biochar's potential benefits relative to current scientific understanding. Our review looked specifically for field studies because they provide unique and essential data for deriving estimates of biochar stability under real-world conditions, and our findings suggest a need for caution with regard to claims about climate-relevant variables, including biochar stability and effects on non-CO_2_ GHG emissions.

## Conclusions

- The study of biochar behavior in soil is a very young field, as reflected in diverse, non-standardized terminology and methods, and uneven distribution of studies across topic areas.
- We need a systematic field research program that investigates stability of biochars representing a range of feedstocks and production methods, across climate and soil gradients.
- We lack the field studies that are needed to understand with confidence how biochar production and application affects whole-system GHG balance. Key variables include, for example, emissions associated with biochar production, transportation, and application to soils; the extent to which biochar amendment stimulates (“primes”) decomposition of soil organic matter; the influence of biochar on non-CO_2_ trace gas emissions; and the amount of energy captured during biochar production.
- The promise and limitations of biochar production and amendment to field soil should be evaluated against a range of biomass management options, including burning biomass for energy and leaving dead wood in place.
- Even with limited available data, it is evident that potential long-term benefits of biochar-based carbon sequestration come at a cost of short-term CO_2_ pulses into the atmosphere and, consequently, near-term acceleration of climate change.
- Optimistic claims about biochar's benefits to the environment contrast sharply with the limited amount of research on biochar's behavior and effects. There is insufficient empirical evidence to support assertions that biochar amendment to soil mitigates climate change significantly, or that it provides overall environmental benefits when evaluated across a comprehensive set of metrics.

## Supporting Information

Figure S1 **PRISMA literature search flow chart for identifying primary research and studies to include in our qualitative synthesis of literature on biochar stability, transport, or fate.**
(PDF)

Figure S2 **Primary research publications.** The numbers correspond to the “Reference Number” in the first column of Figure S3. The 74 citations in bold are publications that addressed the stability, transport, or fate of biochar or soil carbon.
(PDF)

Figure S3 **The topic areas addressed by each primary research publication.** The numbers in the “Reference Number” column correspond to the numbered list in Figure S2.
(XLSX)
