# Supplementary figures and images for: A Systematic Review of Biochar Research, with a Focus on Its Stability in situ and Its Promise as a Climate Mitigation Strategy
Source: PLoS One. 2013 Sep 30;8(9):e75932. doi: 10.1371/journal.pone.0075932 (PMC3786913; doi:10.1371/journal.pone.0075932)

**Figure S1. PRISMA flow diagram for literature search.**

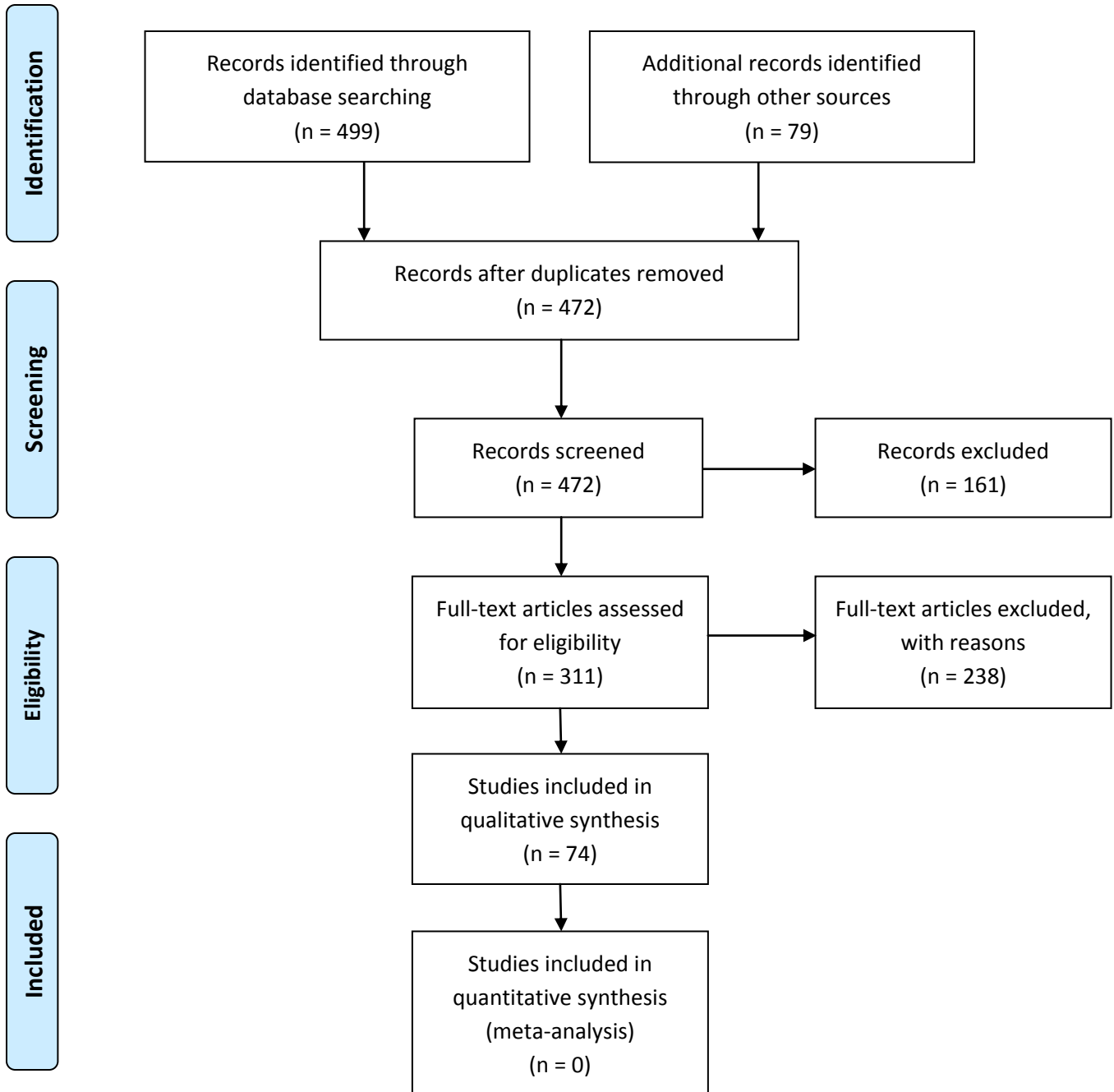

Supplement: Figure S1 — PRISMA literature search flow chart for identifying primary research and studies to include in our qualitative synthesis of literature on biochar stability, transport, or fate. (PDF) [file pone.0075932.s001.pdf]
