# Supplementary material for: A Systematic Review of Biochar Research, with a Focus on Its Stability in situ and Its Promise as a Climate Mitigation Strategy
Source: PLoS One. 2013 Sep 30;8(9):e75932. doi: 10.1371/journal.pone.0075932 (PMC3786913; doi:10.1371/journal.pone.0075932)
Supplement: Figure S2 — Primary research publications. The numbers correspond to the “Reference Number” in the first column of Figure S3. The 74 citations in bold are publications that addressed the stability, transport, or fate of biochar or soil carbon. (PDF) [file pone.0075932.s002.pdf]

Figure S2. Primary research publications. The numbers correspond to the "Reference Number" in the first column of Figure S3. The 74 citations in bold are publications that addressed the stability, transport, or fate of biochar or soil carbon.

1. Abdullah H, Mediaswanti KA, Wu H (2010) Biochar as a Fuel: 2. Significant Differences in Fuel Quality and Ash Properties of Biochars from Various Biomass Components of Mallee Trees. Energy & Fuels 24: 1972-1979.
2. Abdullah H, Wu H (2009) Biochar as a Fuel: 1. Properties and Grindability of Biochars Produced from the Pyrolysis of Mallee Wood under Slow-Heating Conditions. Energy & Fuels 23: 4174-4181.
3. Agblevor FA, Beis S, Kim SS, Tarrant R, Mante NO (2010) Biocrude oils from the fast pyrolysis of poultry litter and hardwood. Waste Management 30: 298-307.
4. Akalin MK, Karagoz S (2011) Pyrolysis of Tobacco Residue: Part 1. Thermal. Bioresources 6: 1520-1531.
5. Akalin MK, Karagoz S (2011) Pyrolysis of Tobacco Residue. Part 2: Catalytic. Bioresources 6: 1773-1805.
6. Alexis M, Rasse D, Rumpel C, Bardoux G, Péchot N, et al. (2007) Fire impact on C and N losses and charcoal production in a scrub oak ecosystem. Biogeochemistry 82: 201-216.
7. **Alexis MA, Bardoux G, Knicker H, Leifeld J, Mariotti A, et al. (2010) Thermal alteration of organic matter during a shrubland fire: A field study. Organic Geochemistry 41: 690-697.**
8. Anderson CR, Condron LM, Clough TJ, Fiers M, Stewart A, et al. (2011) Biochar induced soil microbial community change: Implications for biogeochemical cycling of carbon, nitrogen and phosphorus. Pedobiologia 54: 309-320.
9. Asai H, Samson BK, Stephan HM, Songyikhangsuthor K, Homma K, et al. (2009) Biochar amendment techniques for upland rice production in Northern Laos 1. Soil physical properties, leaf SPAD and grain yield. Field Crops Research 111: 81-84.
10. **Ascough PL, Sturrock CJ, Bird MI (2010) Investigation of growth responses in saprophytic fungi to charred biomass. Isotopes in Environmental and Health Studies 46: 64-77.**
11. Azargohar R, Dalai AK (2006) Biochar as a precursor of activated carbon. Applied Biochemistry and Biotechnology 131: 762-773.
12. Azargohar R, Dalai AK (2008) Steam and KOH activation of biochar: Experimental and modeling studies. Microporous and Mesoporous Materials 110: 413-421.
13. Bailey VL, Fansler SJ, Smith JL, Bolton H (2011) Reconciling apparent variability in effects of biochar amendment on soil enzyme activities by assay optimization. Soil Biology & Biochemistry 43: 296-301.
14. **Baldock JA, Smernik RJ (2002) Chemical composition and bioavailability of thermally altered Pinus resinosa (Red pine) wood. Organic Geochemistry 33: 1093-1109.**
15. **Beck DA, Johnson GR, Spolek GA (2011) Amending greenroof soil with biochar to affect runoff water quantity and quality. Environmental Pollution 159: 2111-2118.**
16. **Beesley L, Dickinson N (2011) Carbon and trace element fluxes in the pore water of an urban soil following greenwaste compost, woody and biochar amendments,**

inoculated with the earthworm Lumbricus terrestris. Soil Biology & Biochemistry 43: 188-196.

17. Beesley L, Marmiroli M (2011) The immobilisation and retention of soluble arsenic, cadmium and zinc by biochar. Environmental Pollution 159: 474-480.
18. Beesley L, Moreno-Jimenez E, Gomez-Eyles JL (2010) Effects of biochar and greenwaste compost amendments on mobility, bioavailability and toxicity of inorganic and organic contaminants in a multi-element polluted soil. Environmental Pollution 158: 2282-2287.
19. **Bell MJ, Worrall F (2011) Charcoal addition to soils in NE England: A carbon sink with environmental co-benefits? Science of the Total Environment 409: 1704-1714.**
20. Bird MI, Ascough PL, Young IM, Wood CV, Scott AC (2008) X-ray microtomographic imaging of charcoal. Journal of Archaeological Science 35: 2698-2706.
21. **Bird MI, Moyo C, Veenendaal EM, Lloyd J, Frost P (1999) Stability of elemental carbon in a savanna soil. Global Biogeochem Cycles 13: 923-932**.
22. Bird MI, Wurster CM, Silva PHD, Bass AM, de Nys R (2011) Algal biochar - production and properties. Bioresource Technology 102: 1886-1891.
23. Blackwell P, Krull E, Butler G, Herbert A, Solaiman Z (2010) Effect of banded biochar on dryland wheat production and fertiliser use in south-western Australia: an agronomic and economic perspective. Soil Research 48: 531-545.
24. Boateng AA (2007) Characterization and thermal conversion of charcoal derived from fluidized-bed fast pyrolysis oil production of switchgrass. Industrial & Engineering Chemistry Research 46: 8857-8862.
25. Boateng AA, Mullen CA, Goldberg NM, Hicks KB, Devine TE, et al. (2010) Sustainable production of bioenergy and biochar from the straw of high-biomass soybean lines via fast pyrolysis. Environmental Progress & Sustainable Energy 29: 175-183.
26. Brewer CE, Schmidt-Rohr K, Satrio JA, Brown RC (2009) Characterization of biochar from fast pyrolysis and gasification systems. Environmental Progress & Sustainable Energy 28: 386-396.
27. Brewer CE, Unger R, Schmidt-Rohr K, Brown RC (2011) Criteria to Select Biochars for Field Studies based on Biochar Chemical Properties. Bioenergy Research 4: 312-323.
28. Brockhoff SR, Christians NE, Killorn RJ, Horton R, Davis DD (2010) Physical and Mineral-Nutrition Properties of Sand-Based Turfgrass Root Zones Amended with Biochar. Agron J 102: 1627-1631.
29. **Brodowski S, Amelung W, Haumaier L, Abetz C, Zech W (2005) Morphological and chemical properties of black carbon in physical soil fractions as revealed by scanning electron microscopy and energy-dispersive X-ray spectroscopy. Geoderma 128: 116-129.**
30. **Brodowski S, Amelung W, Haumaier L, Zech W (2007) Black carbon contribution to stable humus in German arable soils. Geoderma 139: 220-228.**
31. **Brodowski S, John B, Flessa H, Amelung W (2006) Aggregate-occluded black carbon in soil. European Journal of Soil Science 57: 539-546.**
32. Brown TR, Wright MM, Brown RC (2011) Estimating profitability of two biochar production scenarios: slow pyrolysis vs fast pyrolysis. Biofuels Bioproducts & Biorefining-Biofpr 5: 54-68.
33. **Bruun EW, Hauggaard-Nielsen H, Ibrahim N, Egsgaard H, Ambus P, et al. (2011) Influence of fast pyrolysis temperature on biochar labile fraction and short-term carbon loss in a loamy soil. Biomass & Bioenergy 35: 1182-1189.**

34. **Bruun EW, Muller-Stover D, Ambus P, Hauggaard-Nielsen H (2011) Application of biochar to soil and N2O emissions: potential effects of blending fast-pyrolysis biochar with anaerobically digested slurry. European Journal of Soil Science 62: 581-589.**
35. **Bruun S, Jensen ES, Jensen LS (2008) Microbial mineralization and assimilation of black carbon: Dependency on degree of thermal alteration. Organic Geochemistry 39: 839-845.**
36. Bushnaf KM, Puricelli S, Saponaro S, Werner D (2011) Effect of biochar on the fate of volatile petroleum hydrocarbons in an aerobic sandy soil. Journal of Contaminant Hydrology 126: 208-215.
37. **Busscher WJ, Novak JM, Ahmedna M (2011) Physical Effects of Organic Matter Amendment of a Southeastern US Coastal Loamy Sand. Soil Science 176: 661-667.**
38. Busscher WJ, Novak JM, Evans DE, Watts DW, Niandou MAS, et al. (2010) Influence of Pecan Biochar on Physical Properties of a Norfolk Loamy Sand. Soil Science 175: 10-14 10.1097/SS.1090b1013e3181cb1097f1046.
39. Cabrera A, Cox L, Spokas KA, Celis R, Hermosin MC, et al. (2011) Comparative Sorption and Leaching Study of the Herbicides Fluometuron and 4-Chloro-2-methylphenoxyacetic Acid (MCPA) in a Soil Amended with Biochars and Other Sorbents. Journal of Agricultural and Food Chemistry 59: 12550-12560.
40. Cao XD, Harris W (2010) Properties of dairy-manure-derived biochar pertinent to its potential use in remediation. Bioresource Technology 101: 5222-5228.
41. Cao XD, Ma LN, Gao B, Harris W (2009) Dairy-Manure Derived Biochar Effectively Sorbs Lead and Atrazine. Environmental Science & Technology 43: 3285-3291.
42. **Cao XD, Ma LN, Liang Y, Gao B, Harris W (2011) Simultaneous Immobilization of Lead and Atrazine in Contaminated Soils Using Dairy-Manure Biochar. Environmental Science & Technology 45: 4884-4889**.
43. Cao XY, Ro KS, Chappell M, Li YA, Mao JD (2011) Chemical Structures of Swine-Manure Chars Produced under Different Carbonization Conditions Investigated by Advanced Solid-State C-13 Nuclear Magnetic Resonance (NMR) Spectroscopy. Energy & Fuels 25: 388-397.
44. **Castaldi S, Riondino M, Baronti S, Esposito FR, Marzaioli R, et al. (2011) Impact of biochar application to a Mediterranean wheat crop on soil microbial activity and greenhouse gas fluxes. Chemosphere 85: 1464-1471.**
45. Chan KY, Van Zwieten L, Meszaros I, Downie A, Joseph S (2007) Agronomic values of greenwaste biochar as a soil amendment. Australian Journal of Soil Research 45: 629-634.
46. Chan KY, Van Zwieten L, Meszaros I, Downie A, Joseph S (2008) Using poultry litter biochars as soil amendments. Australian Journal of Soil Research 46: 437-444.
47. Chen B, Zhou D, Zhu L (2008) Transitional Adsorption and Partition of Nonpolar and Polar Aromatic Contaminants by Biochars of Pine Needles with Different Pyrolytic Temperatures. Environmental Science & Technology 42: 5137-5143.
48. Chen BL, Chen ZM (2009) Sorption of naphthalene and 1-naphthol by biochars of orange peels with different pyrolytic temperatures. Chemosphere 76: 127-133.
49. Chen BL, Chen ZM, Lv SF (2011) A novel magnetic biochar efficiently sorbs organic pollutants and phosphate. Bioresource Technology 102: 716-723.

50. Chen BL, Yuan MX (2011) Enhanced sorption of polycyclic aromatic hydrocarbons by soil amended with biochar. Journal of Soils and Sediments 11: 62-71.
51. Chen TJ, Deng CJ, Liu RH (2010) Effect of Selective Condensation on the Characterization of Bio-oil from Pine Sawdust Fast Pyrolysis Using a Fluidized-Bed Reactor. Energy & Fuels 24: 6616-6623.
52. Chen Y, Shinogi Y, Taira M (2010) Influence of biochar use on sugarcane growth, soil parameters, and groundwater quality. Australian Journal of Soil Research 48: 526-530.
53. Cheng C-H, Lehmann J, Thies JE, Burton SD (2008) Stability of black carbon in soils across a climatic gradient. J Geophys Res 113: G02027.
54. Cheng C-H, Lehmann J, Thies JE, Burton SD, Engelhard MH (2006) Oxidation of black carbon by biotic and abiotic processes. Organic Geochemistry 37: 1477-1488.
55. Cheng C-h, Lehmann JC, Engelhard MH (2008) Natural Oxidation of Black Carbon in Soils: Changes in Molecular Form and Surface Charge along a Climosequence. Geochimica et Cosmochimica Acta 72: 1598-1610.
56. Chia CH, Munroe P, Joseph S, Lin Y (2010) Microscopic characterisation of synthetic Terra Preta. Soil Research 48: 593-605.
57. Clough TJ, Bertram JE, Ray JL, Condron LM, O'Callaghan M, et al. (2010) Unweathered Wood Biochar Impact on Nitrous Oxide Emissions from a Bovine-Urine-Amended Pasture Soil. Soil Sci Soc Am J 74: 852-860.
58. Cooper JM, Butler G, Leifert C (2011) Life cycle analysis of greenhouse gas emissions from organic and conventional food production systems, with and without bio-energy options. Njas-Wageningen Journal of Life Sciences 58: 185-192.
59. Cross A, Sohi SP (2011) The priming potential of biochar products in relation to labile carbon contents and soil organic matter status. Soil Biology & Biochemistry 43: 2127-2134.
60. Cui HJ, Wang MK, Fu ML, Ci E (2011) Enhancing phosphorus availability in phosphorus-fertilized zones by reducing phosphate adsorbed on ferrihydrite using rice straw-derived biochar. Journal of Soils and Sediments 11: 1135-1141.
61. Cui LQ, Li LQ, Zhang AF, Pan GX, Bao DD, et al. (2011) Biochar amendment greatly reduces rice Cd uptake in a contaminated paddy soil: A two-year field experiment. Bioresources 6: 2605-2618.
62. Cui XY, Jia F, Chen YX, Gan J (2011) Influence of single-walled carbon nanotubes on microbial availability of phenanthrene in sediment. Ecotoxicology 20: 1277-1285.
63. Cui XY, Wang HL, Lou LP, Chen YX, Yu YL, et al. (2009) Sorption and genotoxicity of sediment-associated pentachlorophenol and pyrene influenced by crop residue ash. Journal of Soils and Sediments 9: 604-612.
64. Czimczik CI, Preston CM, Schmidt MWI, Schulze E-D (2003) How surface fire in Siberian Scots pine forests affects soil organic carbon in the forest floor: Stocks, molecular structure, and conversion to black carbon (charcoal). Global Biogeochem Cycles 17: 1020.
65. Czimczik CI, Preston CM, Schmidt MWI, Werner RA, Schulze ED (2002) Effects of charring on mass, organic carbon, and stable carbon isotope composition of wood. Organic Geochemistry 33: 1207-1223.
66. Dai X, Boutton TW, Glaser B, Ansley RJ, Zech W (2005) Black carbon in a temperate mixed-grass savanna. Soil Biology and Biochemistry 37: 1879-1881.

67. Das KC, Singh K, Adolphson R, Hawkins B, Oglesby R, et al. (2010) Steam pyrolysis and catalytic steam reforming of biomass for hydrogen and biochar production. Applied Engineering in Agriculture 26: 137-146.
68. de Jongh WA, Carrier M, Knoetze JH (2011) Vacuum pyrolysis of intruder plant biomasses. Journal of Analytical and Applied Pyrolysis 92: 184-193.
69. Deenik JL, Diarra A, Uehara G, Campbell S, Sumiyoshi Y, et al. (2011) Charcoal Ash and Volatile Matter Effects on Soil Properties and Plant Growth in an Acid Ultisol. Soil Science 176: 336-345.
70. Demirbas A (2006) Production and Characterization of Bio-Chars from Biomass via Pyrolysis. Energy Sources, Part A: Recovery, Utilization, and Environmental Effects 28: 413-422.
71. Demirbaş A, Arslan G, Pehlivan E (2006) Recent Studies on Activated Carbons and Fly Ashes from Turkish Resources. Energy Sources, Part A: Recovery, Utilization, and Environmental Effects 28: 627-638.
72. Demirbas A (2004) Determination of calorific values of bio-chars and pyro-oils from pyrolysis of beech trunkbarks. Journal of Analytical and Applied Pyrolysis 72: 215-219.
73. Demirbas A (2004) Effects of temperature and particle size on bio-char yield from pyrolysis of agricultural residues. Journal of Analytical and Applied Pyrolysis 72: 243-248.
74. Deng J, Wang G, Kuang J, Zhang Y, Luo Y (2009) Pretreatment of agricultural residues for co-gasification via torrefaction. Journal of Analytical and Applied Pyrolysis 86: 331-337.
75. Dias BO, Silva CA, Higashikawa FS, Roig A, Sanchez-Monedero MA (2010) Use of biochar as bulking agent for the composting of poultry manure: Effect on organic matter degradation and humification. Bioresource Technology 101: 1239-1246.
76. Dickens AF, Gelinas Y, Masiello CA, Wakeham S, Hedges JI (2004) Reburial of fossil organic carbon in marine sediments. Nature 427: 336-339.
77. Ding Y, Liu YX, Wu WX, Shi DZ, Yang M, et al. (2010) Evaluation of Biochar Effects on Nitrogen Retention and Leaching in Multi-Layered Soil Columns. Water Air and Soil Pollution 213: 47-55.
78. Dong XL, Ma LNQ, Li YC (2011) Characteristics and mechanisms of hexavalent chromium removal by biochar from sugar beet tailing. Journal of Hazardous Materials 190: 909-915.
79. Downie AE, Van Zwieten L, Smernik RJ, Morris S, Munroe PR (2011) Terra Preta Australis: Reassessing the carbon storage capacity of temperate soils. Agriculture Ecosystems & Environment 140: 137-147.
80. Doydora SA, Cabrera ML, Das KC, Gaskin JW, Sonon LS, et al. (2011) Release of Nitrogen and Phosphorus from Poultry Litter Amended with Acidified Biochar. International Journal of Environmental Research and Public Health 8: 1491-1502.
81. Dumroese RK, Heiskanen J, Englund K, Tervahauta A (2011) Pelleted biochar: Chemical and physical properties show potential use as a substrate in container nurseries. Biomass & Bioenergy 35: 2018-2027.
82. Eckmeier E, Gerlach R, Skjemstad JO, Ehrmann O, Schmidt MWI (2007) Minor changes in soil organic carbon and charcoal concentrations detected in a temperate deciduous forest a year after an experimental slash-and-burn. Biogeosciences 4: 377-383.

83. Elad Y, David DR, Harel YM, Borenshtein M, Ben Kalifa H, et al. (2010) Induction of Systemic Resistance in Plants by Biochar, a Soil-Applied Carbon Sequestering Agent. Phytopathology 100: 913-921.
84. Elmer WH, Pignatello JJ (2011) Effect of Biochar Amendments on Mycorrhizal Associations and Fusarium Crown and Root Rot of Asparagus in Replant Soils. Plant Disease 95: 960-966.
85. Ertaş M, Hakkı Alma M (2010) Pyrolysis of laurel (Laurus nobilis L.) extraction residues in a fixed-bed reactor: Characterization of bio-oil and bio-char. Journal of Analytical and Applied Pyrolysis 88: 22-29.
86. Fellet G, Marchiol L, Delle Vedove G, Peressotti A (2011) Application of biochar on mine tailings: Effects and perspectives for land reclamation. Chemosphere 83: 1262-1267.
87. Foereid B, Lehmann J, Major J (2011) Modeling black carbon degradation and movement in soil. Plant and Soil 345: 223-236.
88. Free HF, McGill CR, Rowarth JS, Hedley MJ (2010) The effect of biochars on maize (Zea mays) germination. New Zealand Journal of Agricultural Research 53: 1-4.
89. Fuertes AB, Arbestain MC, Sevilla M, Macia-Agullo JA, Fiol S, et al. (2010) Chemical and structural properties of carbonaceous products obtained by pyrolysis and hydrothermal carbonisation of corn stover. Australian Journal of Soil Research 48: 618-626.
90. Fushimi C, Wada T, Tsutsumi A (2011) Inhibition of steam gasification of biomass char by hydrogen and tar. Biomass & Bioenergy 35: 179-185.
91. Galinato SP, Yoder JK, Granatstein D (2011) The economic value of biochar in crop production and carbon sequestration. Energy Policy 39: 6344-6350.
92. Gao XP, Wu HW (2011) Biochar as a Fuel: 4. Emission Behavior and Characteristics of PM1 and PM10 from the Combustion of Pulverized Biochar in a Drop-Tube Furnace. Energy & Fuels 25: 2702-2710.
93. Gaskin JW, Speir RA, Harris K, Das KC, Lee RD, et al. (2010) Effect of Peanut Hull and Pine Chip Biochar on Soil Nutrients, Corn Nutrient Status, and Yield. Agron J 102: 623-633.
94. Gaskin JW, Steiner C, Harris K, Das KC, Bibens B (2008) Effect of low-temperature pyrolysis conditions on biochhar for agricultural use. Transactions of the Asabe 51: 2061-2069.
95. Gaunt JL, Lehmann J (2008) Energy Balance and Emissions Associated with Biochar Sequestration and Pyrolysis Bioenergy Production. Environmental Science & Technology 42: 4152-4158.
96. Gell K, van Groenigen JW, Cayuela ML (2011) Residues of bioenergy production chains as soil amendments: Immediate and temporal phytotoxicity. Journal of Hazardous Materials 186: 2017-2025.
97. Gheorghe C, Marculescu C, Badea A, Dinca C, Apostol T. Effect of pyrolysis conditions on bio-char production from biomass; 2009. pp. 239-241.
98. Glaser B, Balashov E, Haumaier L, Guggenberger G, Zech W (2000) Black carbon in density fractions of anthropogenic soils of the Brazilian Amazon region. Organic Geochemistry 31: 669-678.
99. Gomez-Eyles JL, Sizmur T, Collins CD, Hodson ME (2011) Effects of biochar and the earthworm Eisenia fetida on the bioavailability of polycyclic aromatic hydrocarbons and potentially toxic elements. Environmental Pollution 159: 616-622.

100. Graber ER, Harel YM, Kolton M, Cytryn E, Silber A, et al. (2010) Biochar impact on development and productivity of pepper and tomato grown in fertigated soilless media. Plant and Soil 337: 481-496.
101. Graber ER, Tsechansky L, Khanukov J, Oka Y (2011) Sorption, Volatilization, and Efficacy of the Fumigant 1,3-Dichloropropene in a Biochar-Amended Soil. Soil Science Society of America Journal 75: 1365-1373.
102. Grierson S, Strezov V, Ellem G, McGregor R, Herbertson J (2009) Thermal characterisation of microalgae under slow pyrolysis conditions. Journal of Analytical and Applied Pyrolysis 85: 118-123.
103. Grierson S, Strezov V, Shah P (2011) Properties of oil and char derived from slow pyrolysis of Tetraselmis chui. Bioresource Technology 102: 8232-8240.
104. Griffin GJ (2011) The effect of fire retardants on combustion and pyrolysis of sugar-cane bagasse. Bioresource Technology 102: 8199-8204.
105. Grossman JM, O'Neill BE, Tsai SM, Liang BQ, Neves E, et al. (2010) Amazonian Anthrosols Support Similar Microbial Communities that Differ Distinctly from Those Extant in Adjacent, Unmodified Soils of the Same Mineralogy. Microbial Ecology 60: 192-205.
106. Guggenberger G, Rodionov A, Shibistova O, Grabe M, Kasansky OA, et al. (2008) Storage and mobility of black carbon in permafrost soils of the forest tundra ecotone in Northern Siberia. Global Change Biology 14: 1367-1381.
107. Haas TJ, Nimlos MR, Donohoe BS (2009) Real-Time and Post-reaction Microscopic Structural Analysis of Biomass Undergoing Pyrolysis. Energy & Fuels 23: 3810-3817.
108. Haefele SM, Konboon Y, Wongboon W, Amarante S, Maarifat AA, et al. (2011) Effects and fate of biochar from rice residues in rice-based systems. Field Crops Research 121: 430-440.
109. Hale SE, Hanley K, Lehmann J, Zimmerman AR, Cornelissen G (2011) Effects of Chemical, Biological, and Physical Aging As Well As Soil Addition on the Sorption of Pyrene to Activated Carbon and Biochar. Environmental Science & Technology 45: 10445-10453.
110. Hamer U, Marschner B, Brodowski S, Amelung W (2004) Interactive priming of black carbon and glucose mineralisation. Organic Geochemistry 35: 823-830.
111. Hammes K, Smernik RJ, Skjemstad JO, Herzog A, Vogt UF, et al. (2006) Synthesis and characterisation of laboratory-charred grass straw (Oryza saliva) and chestnut wood (Castanea sativa) as reference materials for black carbon quantification. Organic Geochemistry 37: 1629–1633.
112. Hammes K, Torn MS, Lapenas AG, Schmidt MWI (2008) Centennial black carbon turnover observed in a Russian steppe soil. Biogeosciences 5: 1339-1350
113. Hammond J, Shackley S, Sohi S, Brownsort P (2011) Prospective life cycle carbon abatement for pyrolysis biochar systems in the UK. Energy Policy 39: 2646-2655.
114. Hartley W, Dickinson NM, Riby P, Lepp NW (2009) Arsenic mobility in brownfield soils amended with green waste compost or biochar and planted with Miscanthus. Environmental Pollution 157: 2654-2662.
115. Harvey OR, Herbert BE, Rhue RD, Kuo LJ (2011) Metal Interactions at the Biochar-Water Interface: Energetics and Structure-Sorption Relationships Elucidated by Flow Adsorption Microcalorimetry. Environmental Science & Technology 45: 5550-5556.

116. Hilscher A, Knicker H (2011) Carbon and nitrogen degradation on molecular scale of grass-derived pyrogenic organic material during 28 months of incubation in soil. Soil Biology & Biochemistry 43: 261-270.
117. Hilscher A, Knicker H (2011) Degradation of grass-derived pyrogenic organic material, transport of the residues within a soil column and distribution in soil organic matter fractions during a 28month microcosm experiment. Organic Geochemistry 42: 42-54.
118. Hilscher A, Heister K, Siewert C, Knicker H (2009) Mineralisation and structural changes during the initial phase of microbial degradation of pyrogenic plant residues in soil. Organic Geochemistry 40: 332-342.
119. Hina K, Bishop P, Arbestain MC, Calvelo-Pereira R, Macia-Agullo JA, et al. (2010) Producing biochars with enhanced surface activity through alkaline pretreatment of feedstocks. Australian Journal of Soil Research 48: 606-617.
120. Hockaday WC, Grannas AM, Kim S, Hatcher PG (2006) Direct molecular evidence for the degradation and mobility of black carbon in soils from ultrahigh-resolution mass spectral analysis of dissolved organic matter from a fire-impacted forest soil. Organic Geochemistry 37: 501-510.
121. Hockaday WC, Grannas AM, Kim S, Hatcher PG (2007) The transformation and mobility of charcoal in a fire-impacted watershed. Geochimica et Cosmochimica Acta 71: 3432-3445.
122. Hossain MK, Strezov V, Chan KY, Nelson PF (2010) Agronomic properties of wastewater sludge biochar and bioavailability of metals in production of cherry tomato (Lycopersicon esculentum). Chemosphere 78: 1167-1171.
123. Hossain MK, Strezov V, Chan KY, Ziolkowski A, Nelson PF (2011) Influence of pyrolysis temperature on production and nutrient properties of wastewater sludge biochar. Journal of Environmental Management 92: 223-228.
124. Huang WH, Chen BL (2010) Interaction mechanisms of organic contaminants with burned straw ash charcoal. Journal of Environmental Sciences-China 22: 1586-1594.
125. Inyang M, Gao B, Pullammanappallil P, Ding WC, Zimmerman AR (2010) Biochar from anaerobically digested sugarcane bagasse. Bioresource Technology 101: 8868-8872.
126. Inyang MD, Gao B, Ding WC, Pullammanappallil P, Zimmerman AR, et al. (2011) Enhanced Lead Sorption by Biochar Derived from Anaerobically Digested Sugarcane Bagasse. Separation Science and Technology 46: 1950-1956.
127. Jones BEH, Haynes RJ, Phillips IR (2010) Effect of amendment of bauxite processing sand with organic materials on its chemical, physical and microbial properties. Journal of Environmental Management 91: 2281-2288.
128. Jones DL, Edwards-Jones G, Murphy DV (2011) Biochar mediated alterations in herbicide breakdown and leaching in soil. Soil Biology & Biochemistry 43: 804-813.
129. Jones DL, Murphy DV, Khalid M, Ahmad W, Edwards-Jones G, et al. (2011) Short-term biochar-induced increase in soil CO2 release is both biotically and abiotically mediated. Soil Biology & Biochemistry 43: 1723-1731.
130. Kaal J, Brodowski S, Baldock JA, Nierop KGJ, Cortizas AM (2008) Characterisation of aged black carbon using pyrolysis-GC/MS, thermally assisted hydrolysis and methylation (THM), direct and cross-polarisation 13C nuclear magnetic resonance (DP/CP NMR) and the benzenepolycarboxylic acid (BPCA) method. Organic Geochemistry 39: 1415-1426.

131. Kaal J, Rumpel C (2009) Can pyrolysis-GC/MS be used to estimate the degree of thermal alteration of black carbon? Organic Geochemistry 40: 1179-1187.
132. Kameyama K, Shinogi Y, Miyamoto T, Agarie K (2010) Estimation of net carbon sequestration potential with farmland application of bagasse charcoal: life cycle inventory analysis through a pilot sugarcane bagasse carbonisation plant. Australian Journal of Soil Research 48: 586-592.
133. Kammann CI, Linsel S, Gossling JW, Koyro HW (2011) Influence of biochar on drought tolerance of Chenopodium quinoa Willd and on soil-plant relations. Plant and Soil 345: 195-210.
134. Kane E, Hockaday W, Turetsky M, Masiello C, Valentine D, et al. (2010) Topographic controls on black carbon accumulation in Alaskan black spruce forest soils: implications for organic matter dynamics. Biogeochemistry 100: 39-56.
135. Karagöz S (2009) Energy production from the pyrolysis of waste biomasses. International Journal of Energy Research 33: 576-581.
136. Karakoyun N, Kubilay S, Aktas N, Turhan O, Kasimoglu M, et al. (2011) Hydrogel-Biochar composites for effective organic contaminant removal from aqueous media. Desalination 280: 319-325.
137. Karami N, Clemente R, Moreno-Jimenez E, Lepp NW, Beesley L (2011) Efficiency of green waste compost and biochar soil amendments for reducing lead and copper mobility and uptake to ryegrass. Journal of Hazardous Materials 191: 41-48.
138. Karaosmanoglu F, Isigigur-Ergundenler A, Sever A (2000) Biochar from the straw-stalk of rapeseed plant. Energy & Fuels 14: 336-339.
139. Karhu K, Mattila T, Bergstrom I, Regina K (2011) Biochar addition to agricultural soil increased CH4 uptake and water holding capacity - Results from a short-term pilot field study. Agriculture Ecosystems & Environment 140: 309-313.
140. Kasozi GN, Zimmerman AR, Nkedi-Kizza P, Gao B (2010) Catechol and Humic Acid Sorption onto a Range of Laboratory-Produced Black Carbons (Biochars). Environmental Science & Technology 44: 6189-6195.
141. Keiluweit M, Nico PS, Johnson MG, Kleber M (2010) Dynamic Molecular Structure of Plant Biomass-Derived Black Carbon (Biochar). Environmental Science & Technology 44: 1247-1253.
142. Keith A, Singh B, Singh BP (2011) Interactive Priming of Biochar and Labile Organic Matter Mineralization in a Smectite-Rich Soil. Environmental Science & Technology 45: 9611-9618.
143. Khodadad CLM, Zimmerman AR, Green SJ, Uthandi S, Foster JS (2011) Taxa-specific changes in soil microbial community composition induced by pyrogenic carbon amendments. Soil Biology & Biochemistry 43: 385-392.
144. Kim KH, Eom IY, Lee SM, Choi D, Yeo H, et al. (2011) Investigation of physicochemical properties of biooils produced from yellow poplar wood (Liriodendron tulipifera) at various temperatures and residence times. Journal of Analytical and Applied Pyrolysis 92: 2-9.
145. Kim S, Kaplan LA, Benner R, Hatcher PG (2004) Hydrogen-deficient molecules in natural riverine water samples—evidence for the existence of black carbon in DOM. Marine Chemistry 92: 225-234.

146. Kimetu JM, Lehmann J (2010) Stability and stabilisation of biochar and green manure in soil with different organic carbon contents. Australian Journal of Soil Research 48: 577-585.
147. Klasson KT, Uchimiya M, Lima IM, Boihem LL (2011) Feasibility of removing furfurals from sugar solutions using activated biochars made from agricultural residues. Bioresources 6: 3242-3251.
148. Knicker H, Hilscher A, González-Vila FJ, Almendros G (2008) A new conceptual model for the structural properties of char produced during vegetation fires. Organic Geochemistry 39: 935-939.
149. Knoblauch C, Maarifat AA, Pfeiffer EM, Haefele SM (2011) Degradability of black carbon and its impact on trace gas fluxes and carbon turnover in paddy soils. Soil Biology & Biochemistry 43: 1768-1778.
150. Knowles OA, Robinson BH, Contangelo A, Clucas L (2011) Biochar for the mitigation of nitrate leaching from soil amended with biosolids. Science of the Total Environment 409: 3206-3210.
151. Kolb SE, Fermanich KJ, Dornbush ME (2009) Effect of charcoal quantity on microbial biomass and activity in temperate soils. Soil Science Society of America Journal 73: 1173-1181.
152. Kolton M, Harel YM, Pasternak Z, Graber ER, Elad Y, et al. (2011) Impact of Biochar Application to Soil on the Root-Associated Bacterial Community Structure of Fully Developed Greenhouse Pepper Plants. Applied and Environmental Microbiology 77: 4924-4930.
153. Kong HL, He J, Gao YZ, Wu HF, Zhu XZ (2011) Cosorption of Phenanthrene and Mercury(II) from Aqueous Solution by Soybean Stalk-Based Biochar. Journal of Agricultural and Food Chemistry 59: 12116-12123.
154. Kumar S, Loganathan VA, Gupta RB, Barnett MO (2011) An Assessment of U(VI) removal from groundwater using biochar produced from hydrothermal carbonization. Journal of Environmental Management 92: 2504-2512.
155. Kuzyakov Y, Subbotina I, Chen HQ, Bogomolova I, Xu XL (2009) Black carbon decomposition and incorporation into soil microbial biomass estimated by C-14 labeling. Soil Biology & Biochemistry 41: 210-219.
156. Laird D, Fleming P, Wang BQ, Horton R, Karlen D (2010) Biochar impact on nutrient leaching from a Midwestern agricultural soil. Geoderma 158: 436-442.
157. Laird DA, Fleming P, Davis DD, Horton R, Wang BQ, et al. (2010) Impact of biochar amendments on the quality of a typical Midwestern agricultural soil. Geoderma 158: 443-449.
158. Lammirato C, Miltner A, Kaestner M (2011) Effects of wood char and activated carbon on the hydrolysis of cellobiose by beta-glucosidase from Aspergillus niger. Soil Biology & Biochemistry 43: 1936-1942.
159. Lee JW, Kidder M, Evans BR, Paik S, Buchanan AC, et al. (2010) Characterization of Biochars Produced from Cornstovers for Soil Amendment. Environmental Science & Technology 44: 7970-7974.
160. Lehmann J, Skjemstad J, Sohi S, Carter J, Barson M, et al. (2008) Australian climate–carbon cycle feedback reduced by soil black carbon. Nature Geoscience 1: 832-835.
161. Lei HW, Ren SJ, Julson J (2009) The Effects of Reaction Temperature and Time and Particle Size of Corn Stover on Microwave Pyrolysis. Energy & Fuels 23: 3254-3261.

162. Lenton TM, Vaughan NE (2009) The radiative forcing potential of different climate geoengineering options. Atmos Chem Phys 9: 5539-5561.
163. Li D, Hockaday WC, Masiello CA, Alvarez PJJ (2011) Earthworm avoidance of biochar can be mitigated by wetting. Soil Biology & Biochemistry 43: 1732-1737.
164. Liang B, Lehmann J, Sohi SP, Thies JE, O'Neill B, et al. (2010) Black carbon affects the cycling of non-black carbon in soil. Organic Geochemistry 41: 206-213.
165. Liang B, Lehmann J, Solomon D, Sohi S, Thies JE, et al. (2008) Stability of biomass-derived black carbon in soils. Geochimica et Cosmochimica Acta 72: 6069-6078.
166. Lima IM, Boateng AA, Klasson KT (2010) Physicochemical and adsorptive properties of fast-pyrolysis bio-chars and their steam activated counterparts. Journal of Chemical Technology and Biotechnology 85: 1515-1521.
167. Liu WJ, Zeng FX, Jiang H, Zhang XS (2011) Preparation of high adsorption capacity bio-chars from waste biomass. Bioresource Technology 102: 8247-8252.
168. Liu YX, Yang M, Wu YM, Wang HL, Chen YX, et al. (2011) Reducing CH4 and CO2 emissions from waterlogged paddy soil with biochar. Journal of Soils and Sediments 11: 930-939.
169. Liu ZG, Zhang FS (2009) Removal of lead from water using biochars prepared from hydrothermal liquefaction of biomass. Journal of Hazardous Materials 167: 933-939.
170. Lou LP, Wu BB, Wang LN, Luo L, Xu XH, et al. (2011) Sorption and ecotoxicity of pentachlorophenol polluted sediment amended with rice-straw derived biochar. Bioresource Technology 102: 4036-4041.
171. Luo Y, Durenkamp M, De Nobili M, Lin Q, Brookes PC (2011) Short term soil priming effects and the mineralisation of biochar following its incorporation to soils of different pH. Soil Biology & Biochemistry 43: 2304-2314.
172. Maddi B, Viamajala S, Varanasi S (2011) Comparative study of pyrolysis of algal biomass from natural lake blooms with lignocellulosic biomass. Bioresource Technology 102: 11018-11026.
173. Mahinpey N, Murugan P, Mani T, Raina R (2009) Analysis of Bio-Oil, Biogas, and Biochar from Pressurized Pyrolysis of Wheat Straw Using a Tubular Reactor. Energy & Fuels 23: 2736-2742.
174. Major J, Lehmann J, Rondon M, Goodale C (2010) Fate of soil-applied black carbon: downward migration, leaching and soil respiration. Global Change Biology 16: 1366-1379.
175. Major J, Rondon M, Molina D, Riha SJ, Lehmann J (2010) Maize yield and nutrition during 4 years after biochar application to a Colombian savanna oxisol. Plant and Soil 333: 117-128.
176. Mani T, Murugan P, Mahinpey N (2011) Pyrolysis of Oat Straw and the Comparison of the Product Yield to Wheat and Flax Straw Pyrolysis. Energy & Fuels 25: 2803-2807.
177. Mankasingh U, Choi PC, Ragnarsdottir V (2011) Biochar application in a tropical, agricultural region: A plot scale study in Tamil Nadu, India. Applied Geochemistry 26: S218-S221.
178. Matovic D (2011) Biochar as a viable carbon sequestration option: Global and Canadian perspective. Energy 36: 2011-2016.
179. McBeath AV, Smernik RJ (2009) Variation in the degree of aromatic condensation of chars. Organic Geochemistry 40: 1161-1168.

180. Melchior T, Perkins C, Lichty P, Weimer AW, Steinfeld A (2009) Solar-driven biochar gasification in a particle-flow reactor. Chemical Engineering and Processing 48: 1279-1287.
181. Melligan F, Auccaise R, Novotny EH, Leahy JJ, Hayes MHB, et al. (2011) Pressurised pyrolysis of Miscanthus using a fixed bed reactor. Bioresource Technology 102: 3466-3470.
182. Mohan D, Pittman CU, Bricka M, Smith F, Yancey B, et al. (2007) Sorption of arsenic, cadmium, and lead by chars produced from fast pyrolysis of wood and bark during bio-oil production. Journal of colloid and interface science 310: 57-73.
183. Mohan D, Rajput S, Singh VK, Steele PH, Pittman CU (2011) Modeling and evaluation of chromium remediation from water using low cost bio-char, a green adsorbent. Journal of Hazardous Materials 188: 319-333.
184. Mourant D, Wang ZH, He M, Wang XS, Garcia-Perez M, et al. (2011) Mallee wood fast pyrolysis: Effects of alkali and alkaline earth metallic species on the yield and composition of bio-oil. Fuel 90: 2915-2922.
185. Mukherjee A, Zimmerman AR, Harris W (2011) Surface chemistry variations among a series of laboratory-produced biochars. Geoderma 163: 247-255.
186. Mullen CA, Boateng AA, Hicks KB, Goldberg NM, Moreau RA (2010) Analysis and Comparison of Bio-Oil Produced by Fast Pyrolysis from Three Barley Biomass/Byproduct Streams. Energy & Fuels 24: 699-706.
187. Mullen CA, Boateng AA, Goldberg NM, Lima IM, Laird DA, et al. (2010) Bio-oil and bio-char production from corn cobs and stover by fast pyrolysis. Biomass and Bioenergy 34: 67-74.
188. Nag SK, Kookana R, Smith L, Krull E, Macdonald LM, et al. (2011) Poor efficacy of herbicides in biochar-amended soils as affected by their chemistry and mode of action. Chemosphere 84: 1572-1577.
189. Namgay T, Singh B, Singh BP (2010) Influence of biochar application to soil on the availability of As, Cd, Cu, Pb, and Zn to maize (Zea mays L.). Australian Journal of Soil Research 48: 638-647.
190. Nelson NO, Agudelo SC, Yuan WQ, Gan J (2011) Nitrogen and Phosphorus Availability in Biochar-Amended Soils. Soil Science 176: 218-226.
191. Nguyen BT, Lehmann J (2009) Black carbon decomposition under varying water regimes. Organic Geochemistry 40: 846-853.
192. Nguyen BT, Lehmann J, Kinyangi J, Smernik R, Riha SJ, et al. (2009) Long-term black carbon dynamics in cultivated soil. Biogeochemistry 92: 163-176.
193. Ni JZ, Pignatello JJ, Xing BS (2011) Adsorption of Aromatic Carboxylate Ions to Black Carbon (Biochar) Is Accompanied by Proton Exchange with Water. Environmental Science & Technology 45: 9240-9248.
194. Noguera D, Rondon M, Laossi KR, Hoyos V, Lavelle P, et al. (2010) Contrasted effect of biochar and earthworms on rice growth and resource allocation in different soils. Soil Biology & Biochemistry 42: 1017-1027.
195. Novak JM, Busscher WJ, Laird DL, Ahmedna M, Watts DW, et al. (2009) Impact of Biochar Amendment on Fertility of a Southeastern Coastal Plain Soil. Soil Science 174: 105-112.

196. Novak JM, Busscher WJ, Watts DW, Laird DA, Ahmedna MA, et al. (2010) Short-term CO2 mineralization after additions of biochar and switchgrass to a Typic Kandiudult. Geoderma 154: 281-288.
197. Ogawa M, Okimori Y, Takahashi F (2006) Carbon sequestration by carbonization of biomass and forestation: three case studies. Mitigation and Adaptation Strategies for Global Change 11: 421-436.
198. Ohlson M, Dahlberg B, Okland T, Brown KJ, Halvorsen R (2009) The charcoal carbon pool in boreal forest soils. Nature Geosci 2: 692-695.
199. Olarieta JR, Padro R, Masip G, Rodriguez-Ochoa R, Tello E (2011) 'Formiguers', a historical system of soil fertilization (and biochar production?). Agriculture Ecosystems & Environment 140: 27-33.
200. Ozcimen D, Ersoy-Mericboyu A (2008) A study on the carbonization of grapeseed and chestnut shell. Fuel Processing Technology 89: 1041-1046.
201. Ozcimen D, Ersoy-Mericboyu A (2010) Characterization of biochar and bio-oil samples obtained from carbonization of various biomass materials. Renewable Energy 35: 1319-1324.
202. Ozcimen D, Karaosmanoglu F (2004) Production and characterization of bio-oil and biochar from rapeseed cake. Renewable Energy 29: 779-787.
203. Ozyurtkan MH, Ozcimen D, Mericboyu AE (2008) Investigation of the carbonization behavior of hybrid poplar. Fuel Processing Technology 89: 858-863.
204. Park JH, Choppala GK, Bolan NS, Chung JW, Chuasavathi T (2011) Biochar reduces the bioavailability and phytotoxicity of heavy metals. Plant and Soil 348: 439-451.
205. Peng X, Ye LL, Wang CH, Zhou H, Sun B (2011) Temperature- and duration-dependent rice straw-derived biochar: Characteristics and its effects on soil properties of an Ultisol in southern China. Soil & Tillage Research 112: 159-166.
206. Pilon G, Lavoie JM (2011) Characterization of switchgrass char produced in torrefaction and pyrolysis conditions. Bioresources 6: 4824-4839.
207. Potter MC (1908) Bacteria as Agents in the Oxidation of Amorphous Carbon. Proceedings of the Royal Society of London Series B, Containing Papers of a Biological Character 80: 239-259.
208. Pratt K, Moran D (2010) Evaluating the cost-effectiveness of global biochar mitigation potential. Biomass & Bioenergy 34: 1149-1158.
209. Prendergast-Miller MT, Duvall M, Sohi SP (2011) Localisation of nitrate in the rhizosphere of biochar-amended soils. Soil Biology & Biochemistry 43: 2243-2246.
210. Purevsuren B, Avid B, Davaajav Y, Herod AA, Kandiyoti R, et al. (2004) Estimation of the molecular mass range of the tar from pyrolysis of casein by gas chromatography-mass spectrometry, probe mass spectrometry and size-exclusion chromatography with 1-methyl-2-pyrrolidinone as eluent. European Journal of Mass Spectrometry 10: 101-108.
211. Purevsuren B, Avid B, Tesche B, Davaajav Y (2003) A biochar from casein and its properties. Journal of Materials Science 38: 2347-2351.
212. Purevsuren B, Davaajav Y (2001) Investigation on pyrolysis of casein. Journal of Thermal Analysis and Calorimetry 66: 743-748.
213. Purevsuren B, Davaajav Y (2001) Thermal analysis of casein. Journal of Thermal Analysis and Calorimetry 65: 147-152.
214. Purevsuren B, Avid B, Gerelmaa T, Davaajav Y, Morgan TJ, et al. (2004) The characterisation of tar from the pyrolysis of animal bones. Fuel 83: 799-805.

215. Qiu YP, Zheng ZZ, Zhou ZL, Sheng GD (2009) Effectiveness and mechanisms of dye adsorption on a straw-based biochar. Bioresource Technology 100: 5348-5351.
216. Ramsurn H, Kumar S, Gupta RB (2011) Enhancement of Biochar Gasification in Alkali Hydrothermal Medium by Passivation of Inorganic Components Using Ca(OH)(2). Energy & Fuels 25: 2389-2398.
217. Rillig MC, Wagner M, Salem M, Antunes PM, George C, et al. (2010) Material derived from hydrothermal carbonization: Effects on plant growth and arbuscular mycorrhiza. Applied Soil Ecology 45: 238-242.
218. Ro KS, Cantrell KB, Hunt PG (2010) High-Temperature Pyrolysis of Blended Animal Manures for Producing Renewable Energy and Value-Added Biochar. Industrial & Engineering Chemistry Research 49: 10125-10131.
219. Roberts KG, Gloy BA, Joseph S, Scott NR, Lehmann J (2010) Life Cycle Assessment of Biochar Systems: Estimating the Energetic, Economic, and Climate Change Potential. Environmental Science & Technology 44: 827-833.
220. Rogovska N, Laird D, Cruse R, Fleming P, Parkin T, et al. (2011) Impact of Biochar on Manure Carbon Stabilization and Greenhouse Gas Emissions. Soil Science Society of America Journal 75: 871-879.
221. Rondon MA, Lehmann J, Ramirez J, Hurtado M (2007) Biological nitrogen fixation by common beans (Phaseolus vulgaris L.) increases with bio-char additions. Biology and Fertility of Soils 43: 699-708.
222. Saito T, Otani T, Seike N, Murano H, Okazaki M (2011) Suppressive effect of soil application of carbonaceous adsorbents on dieldrin uptake by cucumber fruits. Soil Science and Plant Nutrition 57: 157-166.
223. Salleh MAM, Kisiki NH, Yusuf HM, Ghani W (2010) Gasification of Biochar from Empty Fruit Bunch in a Fluidized Bed Reactor. Energies 3: 1344-1352.
224. Sanchez ME, Lindao E, Margaleff D, Martinez O, Moran A (2009) Pyrolysis of agricultural residues from rape and sunflowers: Production and characterization of bio-fuels and biochar soil management. Journal of Analytical and Applied Pyrolysis 85: 142-144.
225. Sanchez ME, Lindao E, Margaleff D, Martinez O, Moran A (2009) Bio-Fuels and Bio-Char Production from Pyrolysis of Sewage Sludge. Journal of Residuals Science & Technology 6: 35-41.
226. Sanna A, Li SJ, Linforth R, Smart KA, Andresen JM (2011) Bio-oil and bio-char from low temperature pyrolysis of spent grains using activated alumina. Bioresource Technology 102: 10695-10703.
227. Sarmah AK, Srinivasan P, Smernik RJ, Manley-Harris M, Antal MJ, et al. (2010) Retention capacity of biochar-amended New Zealand dairy farm soil for an estrogenic steroid hormone and its primary metabolite. Australian Journal of Soil Research 48: 648-658.
228. Scheer C, Grace PR, Rowlings DW, Kimber S, Van Zwieten L (2011) Effect of biochar amendment on the soil-atmosphere exchange of greenhouse gases from an intensive subtropical pasture in northern New South Wales, Australia. Plant and Soil 345: 47-58.
229. Schmidt MWI, Skjemstad JO, Jäger C (2002) Carbon isotope geochemistry and nanomorphology of soil black carbon: Black chernozemic soils in central Europe originate from ancient biomass burning. Global Biogeochem Cycles 16: 1123.
230. Schneider MPW, Hilf M, Vogt UF, Schmidt MWI (2010) The benzene polycarboxylic acid (BPCA) pattern of wood pyrolyzed between 200 degrees C and 1000 degrees C. Organic Geochemistry 41: 1082-1088.

231. Schwaiger N, Feiner R, Zahel K, Pieber A, Witek V, et al. (2011) Liquid and Solid Products from Liquid-Phase Pyrolysis of Softwood. Bioenergy Research 4: 294-302.
232. Şensöz S, Angın D (2008) Pyrolysis of safflower (Charthamus tinctorius L.) seed press cake: Part 1. The effects of pyrolysis parameters on the product yields. Bioresource Technology 99: 5492-5497.
233. Shroyer KJ, Staggenborg SA, Propheter JL (2011) Utilization of Dry Distillers Grains and Charcoal as Nitrogen Fertilizer in Corn. Agronomy Journal 103: 1321-1328.
234. Silber A, Levkovitch I, Graber ER (2010) pH-Dependent Mineral Release and Surface Properties of Cornstraw Biochar: Agronomic Implications. Environmental Science & Technology 44: 9318-9323.
235. Singh B, Singh BP, Cowie AL (2010) Characterisation and evaluation of biochars for their application as a soil amendment. Australian Journal of Soil Research 48: 516-525.
236. Singh BP, Hatton BJ, Singh B, Cowie AL, Kathuria A (2010) Influence of Biochars on Nitrous Oxide Emission and Nitrogen Leaching from Two Contrasting Soils. Journal of Environmental Quality 39: 1224-1235.
237. Singh N, Kookana RS (2009) Organo-mineral interactions mask the true sorption potential of biochars in soils. Journal of Environmental Science and Health Part B 44: 214-219.
238. Singh N (2008) Sorption of Carbaryl and Ethion on Synthetic Biochars. Pesticide Research Journal 20: 284-286.
239. Sizmur T, Wingate J, Hutchings T, Hodson ME (2011) Lumbricus terrestris L. does not impact on the remediation efficiency of compost and biochar amendments. Pedobiologia 54: S211-S216.
240. Skjemstad JO, Reicosky DC, Wilts AR, McGowan JA (2002) Charcoal Carbon in U.S. Agricultural Soils. Soil Sci Soc Am J 66: 1249-1255.
241. Smith JL, Collins HP, Bailey VL (2010) The effect of young biochar on soil respiration. Soil Biology & Biochemistry 42: 2345-2347.
242. Solaiman ZM, Blackwell P, Abbott LK, Storer P (2010) Direct and residual effect of biochar application on mycorrhizal root colonisation, growth and nutrition of wheat. Australian Journal of Soil Research 48: 546-554.
243. Soria AJ, McDonald AG, Shook SR (2008) Wood solubilization and depolymerization using supercritical methanol. Part 1: Process optimization and analysis of methanol insoluble components (bio-char). Holzforschung 62: 402-408.
244. Spokas K, Reicosky D (2009) Impacts of sixteen different biochars on soil greenhouse gas production. Annals of Environmental Science 3.
245. Spokas KA, Baker JM, Reicosky DC (2010) Ethylene: potential key for biochar amendment impacts. Plant and Soil 333: 443-452.
246. Spokas KA, Koskinen WC, Baker JM, Reicosky DC (2009) Impacts of woodchip biochar additions on greenhouse gas production and sorption/degradation of two herbicides in a Minnesota soil. Chemosphere 77: 574-581.
247. Spokas KA, Novak JM, Stewart CE, Cantrell KB, Uchimiya M, et al. (2011) Qualitative analysis of volatile organic compounds on biochar. Chemosphere 85: 869-882.
248. Steinbeiss S, Gleixner G, Antonietti M (2009) Effect of biochar amendment on soil carbon balance and soil microbial activity. Soil Biology & Biochemistry 41: 1301-1310.
249. Steiner C, Das KC, Melear N, Lakly D (2010) Reducing Nitrogen Loss during Poultry Litter Composting Using Biochar. Journal of Environmental Quality 39: 1236-1242.

250. Steiner C, Glaser B, Teixeira WG, Lehmann J, Blum WEH, et al. (2008) Nitrogen retention and plant uptake on a highly weathered central Amazonian Ferralsol amended with compost and charcoal. Journal of Plant Nutrition and Soil Science-Zeitschrift Fur Pflanzenernahrung Und Bodenkunde 171: 893-899.
251. Streubel JD, Collins HP, Garcia-Perez M, Tarara J, Granatstein D, et al. (2011) Influence of Contrasting Biochar Types on Five Soils at Increasing Rates of Application. Soil Science Society of America Journal 75: 1402-1413.
252. Taghizadeh-Toosi A, Clough TJ, Condron LM, Sherlock RR, Anderson CR, et al. (2011) Biochar Incorporation into Pasture Soil Suppresses in situ Nitrous Oxide Emissions from Ruminant Urine Patches. Journal of Environmental Quality 40: 468-476.
253. Teixido M, Pignatello JJ, Beltran JL, Granados M, Peccia J (2011) Speciation of the Ionizable Antibiotic Sulfamethazine on Black Carbon (Biochar). Environmental Science & Technology 45: 10020-10027.
254. Tong XJ, Li JY, Yuan JH, Xu RK (2011) Adsorption of Cu(II) by biochars generated from three crop straws. Chemical Engineering Journal 172: 828-834.
255. Torri C, Samori C, Adamiano A, Fabbri D, Faraloni C, et al. (2011) Preliminary investigation on the production of fuels and bio-char from Chlamydomonas reinhardtii biomass residue after bio-hydrogen production. Bioresource Technology 102: 8707-8713.
256. Trakal L, Komarek M, Szakova J, Zemanova V, Tlustos P (2011) Biochar application to metal-contaminated soil: Evaluating of Cd, Cu, Pb and Zn sorption behavior using single- and multi-element sorption experiment. Plant Soil and Environment 57: 372-380.
257. Tryon EH (1948) Effect of charcoal on certain physical, chemical, and biological properties of forest soils. Ecological Monographs 18: 81-115.
258. Tsai WT, Tsai YL, Liu SC (2011) Utilization of driftwood as an energy source and its environmental and economic benefit analysis in Taiwan. Bioresources 6: 4781-4789.
259. Uchimiya M, Chang S, Klasson KT (2011) Screening biochars for heavy metal retention in soil: Role of oxygen functional groups. Journal of Hazardous Materials 190: 432-441.
260. Uchimiya M, Klasson KT, Wartelle LH, Lima IM (2011) Influence of soil properties on heavy metal sequestration by biochar amendment: 1. Copper sorption isotherms and the release of cations. Chemosphere 82: 1431-1437.
261. Uchimiya M, Klasson KT, Wartelle LH, Lima IM (2011) Influence of soil properties on heavy metal sequestration by biochar amendment: 2. Copper desorption isotherms. Chemosphere 82: 1438-1447.
262. Uchimiya M, Lima IM, Klasson KT, Chang SC, Wartelle LH, et al. (2010) Immobilization of Heavy Metal Ions (Cu-II, Cd-II, Ni-II, and Pb-II) by Broiler Litter-Derived Biochars in Water and Soil. Journal of Agricultural and Food Chemistry 58: 5538-5544.
263. Uchimiya M, Lima IM, Klasson KT, Wartelle LH (2010) Contaminant immobilization and nutrient release by biochar soil amendment: Roles of natural organic matter. Chemosphere 80: 935-940.
264. Uchimiya M, Wartelle LH, Klasson KT, Fortier CA, Lima IM (2011) Influence of Pyrolysis Temperature on Biochar Property and Function as a Heavy Metal Sorbent in Soil. Journal of Agricultural and Food Chemistry 59: 2501-2510.
265. Uchimiya M, Wartelle LH, Lima IM, Klasson KT (2010) Sorption of Deisopropylatrazine on Broiler Litter Biochars. Journal of Agricultural and Food Chemistry 58: 12350-12356.
266. Unger R, Killorn R (2011) Effect of Three Different Qualities of Biochar on Selected Soil Properties. Communications in Soil Science and Plant Analysis 42: 2274-2283.

267. Unger R, Killorn R (2011) Effect of the Application of Biochar on Selected Soil Chemical Properties, Corn Grain, and Biomass Yields in Iowa. Communications in Soil Science and Plant Analysis 42: 2441-2451.
268. Unger R, Killorn R, Brewer C (2011) Effects of Soil Application of Different Biochars on Selected Soil Chemical Properties. Communications in Soil Science and Plant Analysis 42: 2310-2321.
269. Uzoma KC, Inoue M, Andry H, Fujimaki H, Zahoor A, et al. (2011) Effect of cow manure biochar on maize productivity under sandy soil condition. Soil Use and Management 27: 205-212.
270. Uzoma KC, Inoue M, Andry H, Zahoor A, Nishihara E (2011) Influence of biochar application on sandy soil hydraulic properties and nutrient retention. Journal of Food Agriculture & Environment 9: 1137-1143.
271. Uzun BB, Apaydin-Varol E, Ateş F, Özbay N, Pütün AE (2010) Synthetic fuel production from tea waste: Characterisation of bio-oil and bio-char. Fuel 89: 176-184.
272. Vaccari FP, Baronti S, Lugato E, Genesio L, Castaldi S, et al. (2011) Biochar as a strategy to sequester carbon and increase yield in durum wheat. European Journal of Agronomy 34: 231-238.
273. van Zwieten L, Kimber S, Downie A, Morris S, Petty S, et al. (2010) A glasshouse study on the interaction of low mineral ash biochar with nitrogen in a sandy soil. Australian Journal of Soil Research 48: 569-576.
274. Van Zwieten L, Kimber S, Morris S, Chan KY, Downie A, et al. (2010) Effects of biochar from slow pyrolysis of papermill waste on agronomic performance and soil fertility. Plant and Soil 327: 235-246.
275. van Zwieten L, Kimber S, Morris S, Downie A, Berger E, et al. (2010) Influence of biochars on flux of N2O and CO2 from Ferrosol. Australian Journal of Soil Research 48: 555-568.
276. Wang HL, Lin KD, Hou ZN, Richardson B, Gan J (2010) Sorption of the herbicide terbuthylazine in two New Zealand forest soils amended with biosolids and biochars. Journal of Soils and Sediments 10: 283-289.
277. Wang JY, Zhang M, Xiong ZQ, Liu PL, Pan GX (2011) Effects of biochar addition on N2O and CO2 emissions from two paddy soils. Biology and Fertility of Soils 47: 887-896.
278. Wannapeera J, Fungtammasan B, Worasuwannarak N (2011) Effects of temperature and holding time during torrefaction on the pyrolysis behaviors of woody biomass. Journal of Analytical and Applied Pyrolysis 92: 99-105.
279. Wardle DA, Nilsson M-C, Zackrisson O (2008) Fire-Derived Charcoal Causes Loss of Forest Humus. Science 320: 629.
280. Warnock DD, Mummey DL, McBride B, Major J, Lehmann J, et al. (2010) Influences of non-herbaceous biochar on arbuscular mycorrhizal fungal abundances in roots and soils: Results from growth-chamber and field experiments. Applied Soil Ecology 46: 450-456.
281. Warren GP, Robinson JS, Someus E (2009) Dissolution of phosphorus from animal bone char in 12 soils. Nutrient Cycling in Agroecosystems 84: 167-178.
282. Whitman T, Nicholson CF, Torres D, Lehmann J (2011) Climate Change Impact of Biochar Cook Stoves in Western Kenyan Farm Households: System Dynamics Model Analysis. Environmental Science & Technology 45: 3687-3694.

283. Woolf D, Amonette JE, Street-Perrott FA, Lehmann J, Joseph S (2010) Sustainable biochar to mitigate global climate change. Nature Communications 1.
284. Wu HW, Yip K, Kong ZY, Li CZ, Liu DW, et al. (2011) Removal and Recycling of Inherent Inorganic Nutrient Species in Mal lee Biomass and Derived Biochars by Water Leaching. Industrial & Engineering Chemistry Research 50: 12143-12151.
285. Wu HW, Yip K, Tian FJ, Xie ZL, Li CZ (2009) Evolution of Char Structure during the Steam Gasification of Biochars Produced from the Pyrolysis of Various Mallee Biomass Components. Industrial & Engineering Chemistry Research 48: 10431-10438.
286. Xiao XY, Sheng GD, Qiu YP (2011) Improved understanding of tributyltin sorption on natural and biochar-amended sediments. Environmental Toxicology and Chemistry 30: 2682-2687.
287. Xu R, Ferrante L, Hall K, Briens C, Berruti F (2011) Thermal self-sustainability of biochar production by pyrolysis. Journal of Analytical and Applied Pyrolysis 91: 55-66.
288. Yan QG, Toghiani H, Yu F, Cal ZY, Zhang JL (2011) Effects of Pyrolysis Conditions on Yield of Bio-Chars from Pine Chips. Forest Products Journal 61: 367-371.
289. Yang XB, Ying GG, Peng PA, Wang L, Zhao JL, et al. (2010) Influence of Biochars on Plant Uptake and Dissipation of Two Pesticides in an Agricultural Soil. Journal of Agricultural and Food Chemistry 58: 7915-7921.
290. Yao FX, Arbestain MC, Virgel S, Blanco F, Arostegui J, et al. (2010) Simulated geochemical weathering of a mineral ash-rich biochar in a modified Soxhlet reactor. Chemosphere 80: 724-732.
291. Yao Y, Gao B, Inyang M, Zimmerman AR, Cao XD, et al. (2011) Biochar derived from anaerobically digested sugar beet tailings: Characterization and phosphate removal potential. Bioresource Technology 102: 6273-6278.
292. Yao Y, Gao B, Inyang M, Zimmerman AR, Cao XD, et al. (2011) Removal of phosphate from aqueous solution by biochar derived from anaerobically digested sugar beet tailings. Journal of Hazardous Materials 190: 501-507.
293. Yip K, Tian FJ, Hayashi J, Wu HW (2010) Effect of Alkali and Alkaline Earth Metallic Species on Biochar Reactivity and Syngas Compositions during Steam Gasification. Energy & Fuels 24: 173-181.
294. Yip KV, Xu MH, Li CZ, Jiang SP, Wu HW (2011) Biochar as a Fuel: 3. Mechanistic Understanding on Biochar Thermal Annealing at Mild Temperatures and Its Effect on Biochar Reactivity. Energy & Fuels 25: 406-414.
295. Yoder J, Galinato S, Granatstein D, Garcia-Perez M (2011) Economic tradeoff between biochar and bio-oil production via pyrolysis. Biomass & Bioenergy 35: 1851-1862.
296. Yu XY, Mu CL, Gu C, Liu C, Liu XJ (2011) Impact of woodchip biochar amendment on the sorption and dissipation of pesticide acetamiprid in agricultural soils. Chemosphere 85: 1284-1289.
297. Yu XY, Pan LG, Ying GG, Kookana RS (2010) Enhanced and irreversible sorption of pesticide pyrimethanil by soil amended with biochars. Journal of Environmental Sciences-China 22: 615-620.
298. Yu XY, Ying GG, Kookana RS (2009) Reduced plant uptake of pesticides with biochar additions to soil. Chemosphere 76: 665-671.
299. Yu Y, Wu HW (2010) Bioslurry as a Fuel. 2. Life-Cycle Energy and Carbon Footprints of Bioslurry Fuels from Mallee Biomass in Western Australia. Energy & Fuels 24: 5660-5668.

300. Yuan JH, Xu RK (2011) The amelioration effects of low temperature biochar generated from nine crop residues on an acidic Ultisol. Soil Use and Management 27: 110-115.
301. Yuan JH, Xu RK, Qian W, Wang RH (2011) Comparison of the ameliorating effects on an acidic ultisol between four crop straws and their biochars. Journal of Soils and Sediments 11: 741-750.
302. Yuan JH, Xu RK, Wang N, Li JY (2011) Amendment of Acid Soils with Crop Residues and Biochars. Pedosphere 21: 302-308.
303. Yuan JH, Xu RK, Zhang H (2011) The forms of alkalis in the biochar produced from crop residues at different temperatures. Bioresource Technology 102: 3488-3497.
304. Zabaniotou A, Ioannidou O, Antonakou E, Lappas A (2008) Experimental study of pyrolysis for potential energy, hydrogen and carbon material production from lignocellulosic biomass. International journal of hydrogen energy 33: 2433-2444.
305. Zavalloni C, Alberti G, Biasiol S, Delle Vedove G, Fornasier F, et al. (2011) Microbial mineralization of biochar and wheat straw mixture in soil: A short-term study. Applied Soil Ecology 50: 45-51.
306. Zhang AF, Cui LQ, Pan GX, Li LQ, Hussain Q, et al. (2010) Effect of biochar amendment on yield and methane and nitrous oxide emissions from a rice paddy from Tai Lake plain, China. Agriculture Ecosystems & Environment 139: 469-475.
307. Zhang HH, Lin KD, Wang HL, Gan J (2010) Effect of Pinus radiata derived biochars on soil sorption and desorption of phenanthrene. Environmental Pollution 158: 2821-2825.
308. Zhang W, Niu JZ, Morales VL, Chen XC, Hay AG, et al. (2010) Transport and retention of biochar particles in porous media: effect of pH, ionic strength, and particle size. Ecohydrology 3: 497-508.
309. Zheng W, Guo MX, Chow T, Bennett DN, Rajagopalan N (2010) Sorption properties of greenwaste biochar for two triazine pesticides. Journal of Hazardous Materials 181: 121-126.
310. Zimmerman AR (2010) Abiotic and Microbial Oxidation of Laboratory-Produced Black Carbon (Biochar). Environmental Science & Technology 44: 1295-1301.
311. Zimmerman AR, Gao B, Ahn MY (2011) Positive and negative carbon mineralization priming effects among a variety of biochar-amended soils. Soil Biology & Biochemistry 43: 1169-1179.
